# The *Coxiella burnetii* T4SS effector protein AnkG hijacks the 7SK small nuclear ribonucleoprotein complex for reprogramming host cell transcription

**DOI:** 10.1371/journal.ppat.1010266

**Published:** 2022-02-08

**Authors:** Arne Cordsmeier, Sven Rinkel, Myriam Jeninga, Jan Schulze-Luehrmann, Martha Ölke, Benedikt Schmid, Daniele Hasler, Gunter Meister, Georg Häcker, Michaela Petter, Paul A. Beare, Anja Lührmann

**Affiliations:** 1 Mikrobiologisches Institut, Universitätsklinikum Erlangen, Friedrich-Alexander-Universität Erlangen-Nürnberg, Erlangen, Germany; 2 Lehrstuhl für Biotechnik, Department Biologie, Friedrich-Alexander-Universität Erlangen-Nürnberg, Erlangen, Germany; 3 Biochemistry Center Regensburg (BZR), Laboratory for RNA Biology, University of Regensburg, Regensburg, Germany; 4 Faculty of Medicine, Institute of Medical Microbiology and Hygiene, Medical Center-University of Freiburg, Freiburg, Germany; 5 Coxiella Pathogenesis Section, Laboratory of Bacteriology, Rocky Mountain Laboratories, National Institute of Allergy and Infectious Diseases, National Institutes of Health, Hamilton, Montana, United States of America; University of São Paulo FMRP/USP, BRAZIL

## Abstract

Inhibition of host cell apoptosis is crucial for survival and replication of several intracellular bacterial pathogens. To interfere with apoptotic pathways, some pathogens use specialized secretion systems to inject bacterial effector proteins into the host cell cytosol. One of these pathogens is the obligate intracellular bacterium *Coxiella burnetii*, the etiological agent of the zoonotic disease Q fever. In this study, we analyzed the molecular activity of the anti-apoptotic T4SS effector protein AnkG (CBU0781) to understand how *C*. *burnetii* manipulates host cell viability. We demonstrate by co- and RNA-immunoprecipitation that AnkG binds to the host cell DExD box RNA helicase 21 (DDX21) as well as to the host cell 7SK small nuclear ribonucleoprotein (7SK snRNP) complex, an important regulator of the positive transcription elongation factor b (P-TEFb). The co-immunoprecipitation of AnkG with DDX21 is probably mediated by salt bridges and is independent of AnkG-7SK snRNP binding, and *vice versa*. It is known that DDX21 facilitates the release of P-TEFb from the 7SK snRNP complex. Consistent with the documented function of released P-TEFb in RNA Pol II pause release, RNA sequencing experiments confirmed AnkG-mediated transcriptional reprogramming and showed that expression of genes involved in apoptosis, trafficking, and transcription are influenced by AnkG. Importantly, DDX21 and P-TEFb are both essential for AnkG-mediated inhibition of host cell apoptosis, emphasizing the significance of the interaction of AnkG with both, the DDX21 protein and the 7SK RNA. In line with a critical function of AnkG in pathogenesis, the AnkG deletion *C*. *burnetii* strain was severely affected in its ability to inhibit host cell apoptosis and to generate a replicative *C*. *burnetii*-containing vacuole. In conclusion, the interference with the activity of regulatory host cell RNAs mediated by a bacterial effector protein represent a novel mechanism through which *C*. *burnetii* modulates host cell transcription, thereby enhancing permissiveness to bacterial infection.

## Author summary

For intracellular replication, *Coxiella burnetii* depends on a functional type IV secretion system, which is utilized to inject ~150 virulence factors, so called effector proteins, into the host cell cytosol. Activities have only been established for few of them. These effector proteins interfere with vesicular trafficking, autophagy, lipid metabolism, apoptosis, and transcription by binding and manipulating the activity of host cell proteins. Here, we report that the *C*. *burnetii* T4SS effector protein AnkG (CBU0781, Q83DF6) binds to the host cell DExD box helicase 21 (DDX21) as well as to several host cell RNAs, including the small regulatory 7SK RNA, which is an important regulator of the positive elongation factor b (pTEFb). AnkG interferes with the function of the 7SK small nuclear ribonucleoprotein (7SK snRNP) complex, leading to significant changes in host cell transcription and ensuring host cell survival. AnkG activity is essential for efficient intracellular replication of *C*. *burnetii* and its ability to inhibit apoptosis. In summary, we identified a novel process by which a bacterial effector protein manipulates the host cell for its own benefit.

## Introduction

*Coxiella burnetii* is an obligate intracellular pathogen with worldwide distribution, with the exception of New Zealand [1]. It infects multiple vertebrate and invertebrate hosts. However, critical for human infections are mainly infected ruminants, like cattle, sheep, and goats [2]. Infection of ruminants can be asymptomatic or lead to abortion, premature delivery, stillbirth, or weak offspring. Infected animals shed the pathogen through birth products, milk, wool and manure, and these are all possible sources for human infection [3]. Human infection occurs mainly by inhalation of contaminated aerosols [2]. The proportion of symptomatic infections increases with infection dosage, with less than 6 bacteria causing illness in 50% of cases [4], demonstrating the importance to prevent the contamination of the environment with *C*. *burnetii*. In the majority of infected individuals, the infection remains asymptomatic. In other cases, the infection manifests as a flu-like illness, pneumonia or hepatitis. However, the severity of the primary infection is not predictive for the development of later complications, such as post-Q fever fatigue syndrome or chronic Q fever [5,6]. The most frequent manifestation of chronic Q fever is endocarditis with potentially fatal outcome. The development of chronic Q fever endocarditis is in almost all cases associated with an underlying valvulopathy [7]. To treat chronic Q fever a combination of doxycycline and hydroxychloroquine for 18–24 month is recommended. This long treatment comes with severe side effects and reduced compliance of the patients [5]. Thus, we must increase the knowledge about this pathogen, its pathogenicity and disease progression in order to develop new therapeutic strategies.

Alveolar macrophages are the first target cells taking up *C*. *burnetii* after inhalation of contaminated aerosols. Following internalization into the phagosome, the *C*. *burnetii*-containing vacuole (CCV) matures along the endocytic pathway into a phagolysosomal compartment [8]. The low pH within the CCV promotes replication of *C*. *burnetii* [9] and triggers activation of the type IV secretion system (T4SS) [10]. The T4SS is essential for injecting bacterial effector proteins into the host cell cytosol [11]. *C*. *burnetii* lacking a functional T4SS fail to replicate intracellularly [12,13], demonstrating the importance of the T4SS for bacterial virulence. The identified ~150 *C*. *burnetii* T4SS effector proteins modulate different host cell pathways, including host transcription and signaling, vesicular trafficking and host cell viability [8]. However, we are far from understanding their molecular activity, which would open new avenues to develop novel diagnostic or therapeutic tools.

**Fig 1:**
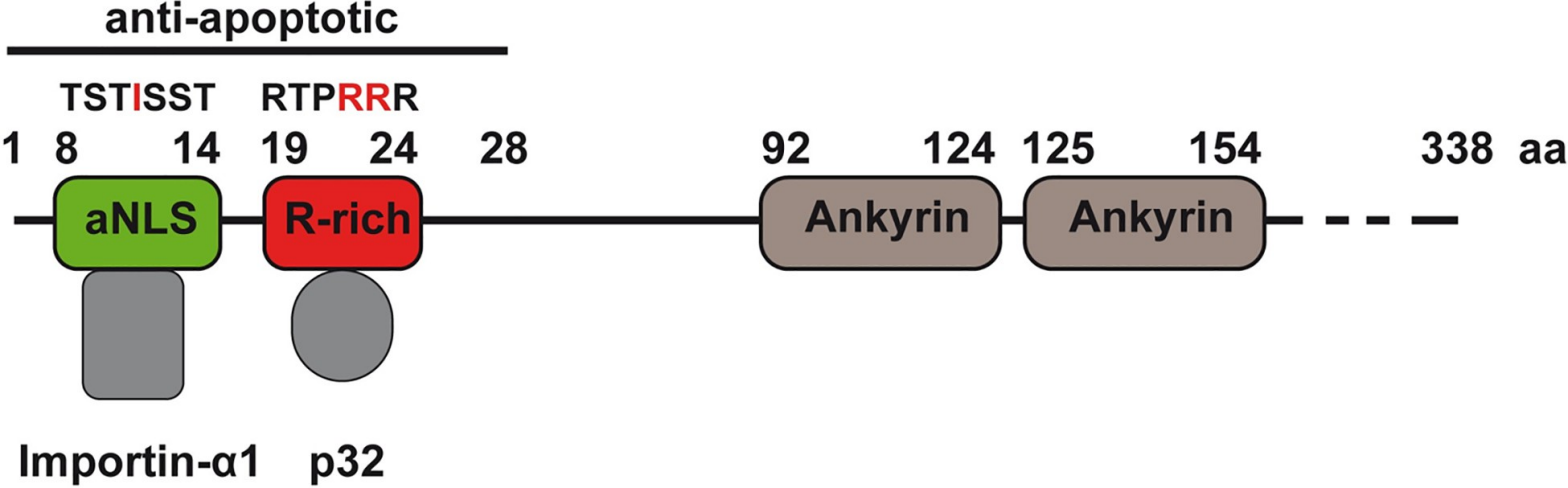
Schematic structure of AnkG. AnkG is a 338 amino acid protein and contains two ankyrin repeat domains (amino acid 92–124 and 125–154). The anti-apoptotic region comprises amino acids 1–28. The amino acids 8–14 contain an atypical nuclear localization signal (aNLS) and interact with Importin-α1. Mutation at the amino acid 11 (isoleucine) abolishes this interaction. The amino acids 19–24 are arginine rich (R-rich) and mediate the interaction with p32. Mutation of the amino acids 23 and 24 prevent the p32 binding.

Here, we focus on the T4SS effector protein AnkG, which has anti-apoptotic activity [14–17]. AnkG´s activity inside the host cell is controlled by intracellular trafficking and depends on nuclear localization [14,16]. The first 28 amino acids are necessary and sufficient for its anti-apoptotic activity (Fig 1) [17]. However, how AnkG alters nuclear activity to prevent host cell death was an open question [18]. AnkG belongs to the group of bacterial nuclear-targeted effector proteins. Many pathogens possess such nucleomodulins, which influence the host cell nucleus [19]. Nucleomodulins might influence the homeostasis of nuclear proteins, act as chromatin-modifying enzymes, trigger post-transcriptional modification or activity of nuclear regulators [20]. Our results demonstrate that AnkG modulates host cell transcription by influencing the activity of the 7SK small ribonucleoprotein (snRNP) complex, which is a critical regulator of RNA polymerase II mediated transcription [21].

## Results

### AnkG interacts with the RNA helicase DDX21

**Fig 2:**
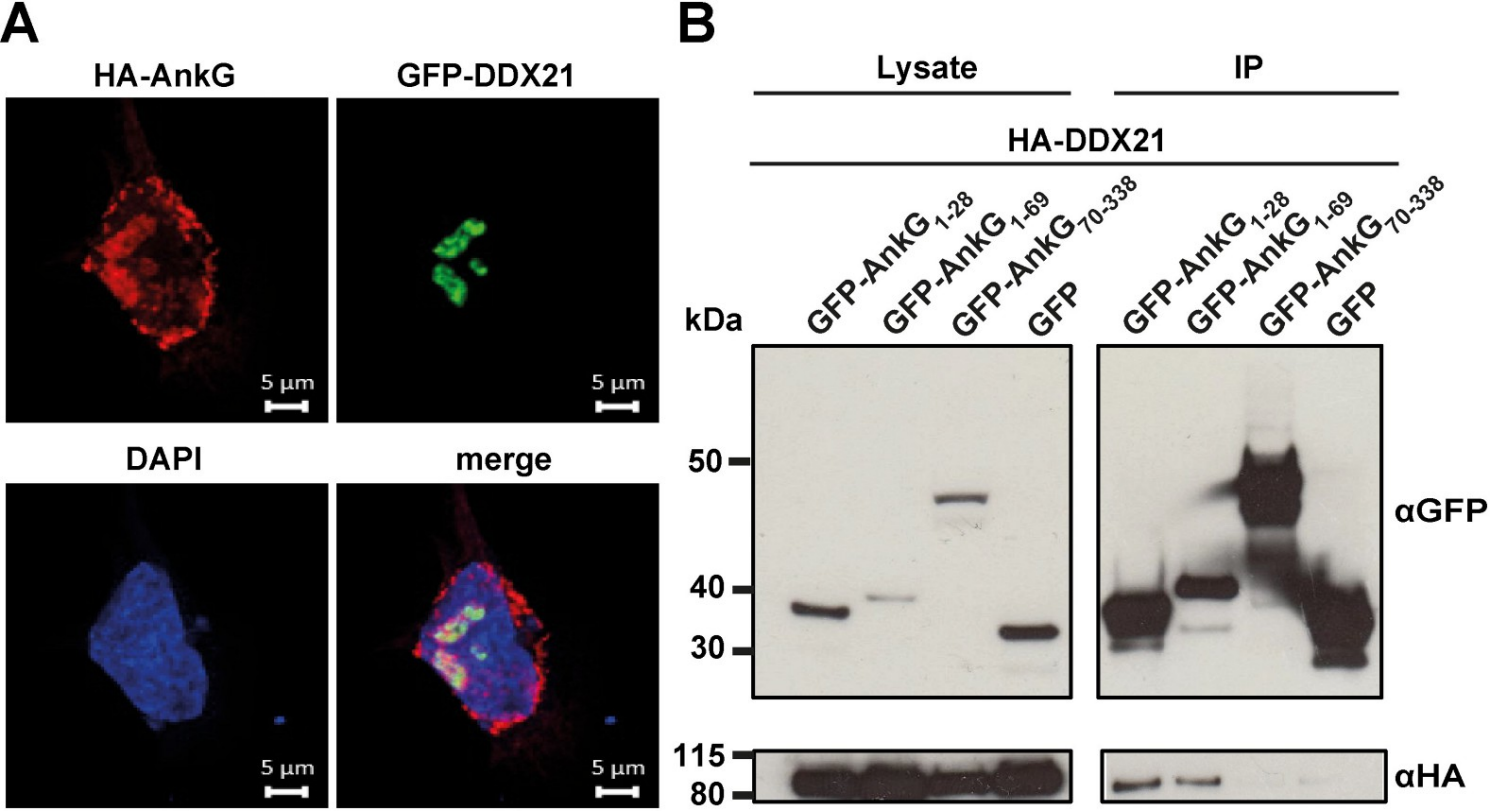
The anti-apoptotic N-terminus of AnkG interacts with DDX21. (A) Representative immunofluorescence images show a HeLa cell transfected with HA-AnkG (red) and GFP-DDX21 (green). (B) HEK293T cells were transiently co-transfected with GFP-tagged AnkG-variants and HA-DDX21. Proteins were precipitated using GFP-trap. Western blot analysis was used to detect DDX21 (anti-HA) and GFP or GFP-tagged AnkG variants (anti-GFP) in the lysates and in the precipitates (IP). A representative western blot out of three independent experiments with similar results is depicted.

AnkG belongs to the nuclear-targeted effector protein group that modulate host cell nuclear processes [19]. In order to understand how AnkG facilitates anti-apoptotic activity we aimed to identify host nuclear proteins as AnkG binding partners. We have previously performed GFP-trap experiments from HEK293T cells ectopically expressing GFP or GFP-AnkG followed by mass spec analysis and identified several nuclear proteins as potential AnkG binding partners [16]. We tested the following top candidates for interaction: hnRNPM, TSR1, transcription repressor p66beta, PRP19, Cdc5L, DDX21 and MTA2. We could confirm co-immunoprecipitation of AnkG with hnRNPM, TSR1, Cdc5L, DDX21 and MTA2 by GFP-trap (S1 Fig). To confirm interaction of AnkG with the respective nuclear proteins, we analyzed the subcellular localization in HeLa cells. We used HeLa cells as they are more suitable for immunofluorescence analysis and to verify that the interaction is not limited to HEK293T cells. As shown in Fig 2A, AnkG co-localizes with DDX21 within substructures of the nucleus. Therefore, we concentrated our analysis on the AnkG-DDX21 interaction. DDX21 is a human DEAD-box RNA helicase that promotes ribosomal RNA (rRNA) processing and transcription from polymerase II (Pol II) [22]. As DDX21 is mainly found in the nucleoli [23], we conclude that the substructures of AnkG-DDX21 co-localization might represent the nucleoli. To get a first impression whether the interaction of AnkG with DDX21 might be important for the anti-apoptotic activity of AnkG, we asked which parts of AnkG might interact with DDX21. We have previously shown that the N-terminal region of AnkG, comprising amino acids 1–28, is necessary and sufficient for anti-apoptotic activity and exclusively localizes in the host cell nucleus, while the region from amino acids 70–338 did not prevent cell death and has a cytoplasmic localization [14,15,17]. As shown in Fig 2B, HA-tagged DDX21 was bound by GFP-AnkG_1-28_ and GFP-AnkG_1-69_, but not by GFP-AnkG_70-338_, indicating that DDX21 binds to the anti-apoptotic region of AnkG.

### AnkG influences subcellular localization of DDX21 under cell stress conditions

**Fig 3:**
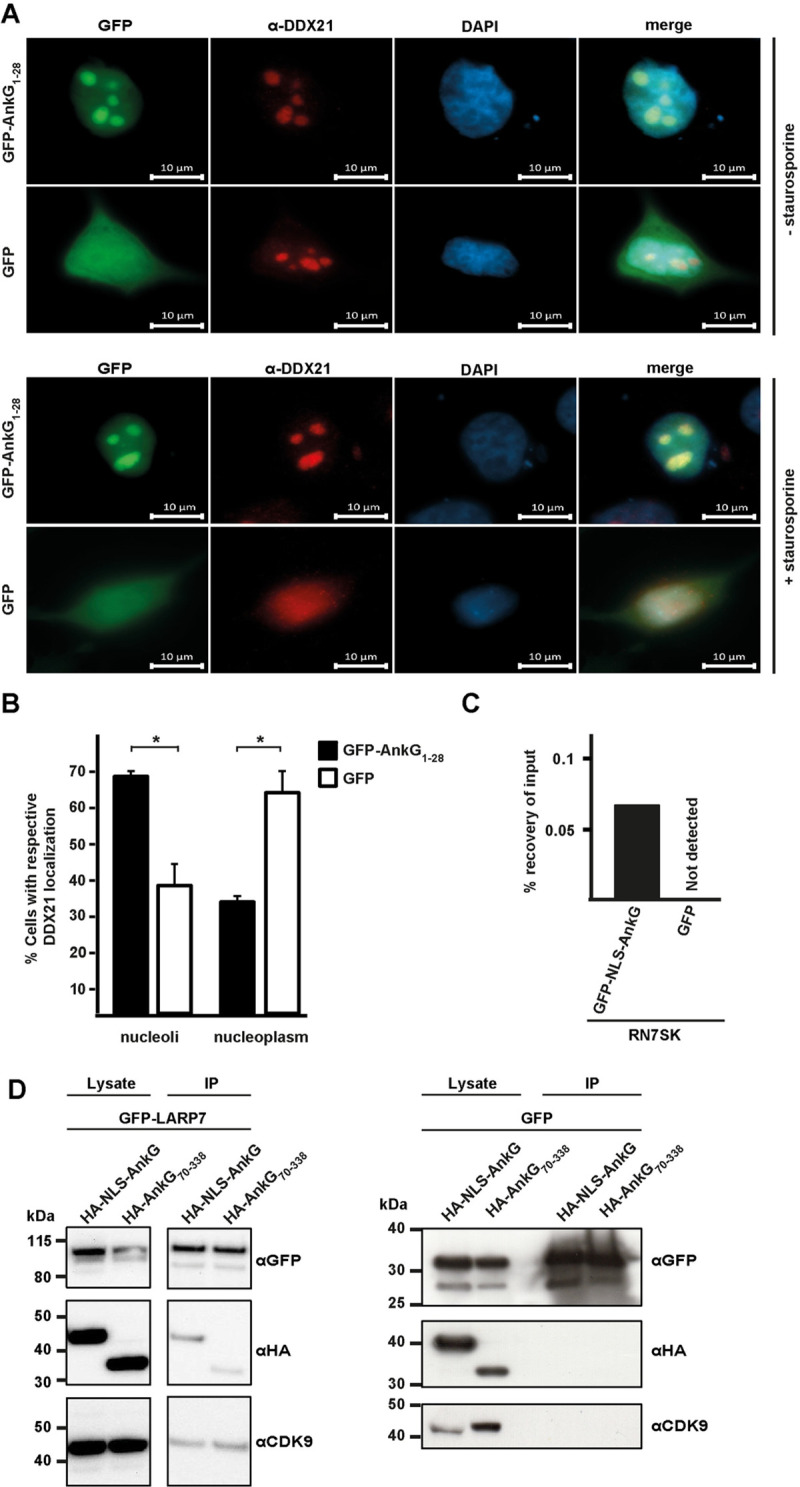
AnkG modulates subcellular localization of DDX21 and interacts with the 7SK snRNP. (A and B) GFP or GFP-AnkG_1-28_ were transiently expressed in HeLa cells. The cells were either not treated or treated for 4 hours with 0.1 μM staurosporine and stained with an antibody against endogenous DDX21. The subcellular localization of DDX21 was analyzed by epifluorescence microscopy. (A) A representative immunofluorescence image is shown. Upper rows without treatment, lower rows with staurosporine-treatment. (B) The DDX21 localization was analyzed from 50 GFP-expressing cells treated with staurosporine in each of three independent experiments. Error bars indicate ± SD. * *p*< 0.05. (C) HEK293T cells were transfected with plasmids encoding GFP or GFP-NLS-AnkG. RNA-immunoprecipitation was performed using an anti-GFP antibody. The 7SK RNA was reverse transcribed and a qRT-PCR was performed using specific primers. Data presented as percentage of input bound by GFP or GFP-NLS-AnkG (n = 3). (D) HEK293T cells were co-transfected with HA-tagged AnkG variants and GFP-LARP7 or GFP as control. Proteins were precipitated using GFP-trap. Western blot analysis was used to detect AnkG variants (anti-HA), GFP-LARP7 or GFP (anti-GFP) and endogenous CDK9 (anti-CDK9) in the lysates and in the precipitates (IP). Representative immunoblots out of three independent experiments with similar results are depicted.

The fact that the anti-apoptotic region of AnkG interacts with DDX21 lead us to hypothesize that AnkG modulates DDX21 to facilitate anti-apoptotic activity. As DDX21 translocates from the nucleolus to the nucleoplasm under pro-apoptotic conditions [24], we examined whether the expression of AnkG influences this trafficking. We could confirm that cell death induction by staurosporine leads to translocation of DDX21 from the nucleolus into the nucleoplasm in HeLa cells (Fig 3A). Importantly, the expression of AnkG_1-28_ prevents this migration and keeps DDX21 within the nucleolus even under pro-apoptotic conditions (Fig 3A and 3B), demonstrating that AnkG modulates DDX21 localization under stress conditions. However, whether this activity is essential for the anti-apoptotic activity of AnkG or is an indirect effect of cell death prevention by AnkG had to be clarified. To this end, we analyzed whether AnkG modulates other known activities of DDX21.

### AnkG binds the 7SK RNA

DDX21 binds to a diverse group of RNAs [22]. Therefore, we aimed to identify RNAs interacting with AnkG in an unbiased way. RNA-protein complexes from HEK293T cells expressing either GFP or GFP-NLS-AnkG were immune-precipitated with an anti-GFP antibody (RIP). We used GFP-NLS-AnkG because due to the nuclear localization sequence (NLS) the nuclear localization of AnkG was ensured. We obtained strongly enriched RNA precipitates from cells expressing GFP-NLS-AnkG, but not from cells expressing GFP. This data suggests that AnkG interacts with RNA or RNA-protein (RNP) complexes. Next, we performed RNAseq of the precipitates to identify the RNAs interacting with GFP-NLS-AnkG. We identified more than 10,000 different RNAs (geo accession number: GSE185428, S1 Table, the most enriched RNAs are shown in Table 1). Importantly, the FPKM values of the top 10 candidates sum up to over 80% of the total FPKM of all identified RNAs, indicating that the majority of identified RNAs are probably background and not strong interaction partners of AnkG. One of the potential AnkG-interacting RNAs with a high FPKM value is the 7SK RNA, which was shown to bind to DDX21 [22]. We confirmed the interaction of GFP-NLS-AnkG with the 7SK RNA by an RNA-immunoprecipitation (RIP) experiment followed by qRT-PCR (Fig 3C). However, only a fraction of identified RNAs that interact with AnkG have previously been shown to also interact with DDX21 (Table 1), suggesting that AnkG binds to RNAs or RNPs independent of binding to DDX21. Additional experiments are required to dissect these interactions and clarify if the AnkG-RNA interaction is direct or mediated by other RNA-binding proteins.

**Table 1:** RNAs identified in the RIP-Seq analysis. The average fragments per kilobase million (FPKM) from three independent experiments are shown. The 15 genes with most enriched RNAs and five additional RNAs known or predicted to interact with DDX21 are depicted. RNAs with confirmed interaction with AnkG are high-lighted in bold letters and interaction with DDX21 (deduced from RNAct database) is indicated in italic letters.

Gene	Name	FPKM	Kind
**CTD-2328D6.1**	-	827162	ncRNA
***RN7SK***	7SK small nuclear	108110	snRNA
**RN7SL1**	7SL RNA	12885	scRNA
RN7SL2	7SL, cytoplasmic 2	5900	scRNA
AC010970.2	-	5187	Processed Pseudogene
**RNU4-1**	U4 small nuclear 1	3782	snRNA
AL161626.1	-	2183	rRNA Pseudogene
RPS2	ribosomal protein S2	1393	coding
Metazoa_SRP	Metazoan signal recognition particle RNA	972	ncRNA
XIST	inactive X specific transcripts	911	ncRNA
BCYRN1	brain cytoplasmic RNA 1	900	ncRNA
PHB2	prohibitin 2	884	coding
RP5-940J5.9	-	839	ncRNA
EEF2	eukaryotic translation elongation factor 2	776	coding
RPS6	ribosomal protein S6	706	coding
*EIF4A1*	eukaryotic translation initiation factor 4A1	315	coding
*RPL7A*	ribosomal protein L7a	244	coding
*GNAS*	GNAS complex locus	233	coding
*RPS20*	ribosomal protein S20	176	coding
*RPL23A*	ribosomal protein L23a	175	coding

### AnkG binds to the 7SK snRNP complex

The 7SK small nuclear ribonucleoprotein (snRNP) complex is composed of the scaffolding 7SK RNA, a 331 nucleotide-long non-coding RNA, the RNA stability protein LARP7, the kinase inhibitor HEXIM1/HEXIM2, the RNA methyl-capping enzyme (MePCE), and the positive transcription elongation factor (P-TEF) b, which is a heterodimer formed of the cyclin dependent kinase (CDK) 9 and either CyclinT1 or CyclinT2. CDK9 is involved in global regulation of gene transcription under basal, but also under activating (stress), conditions. Thus, it is important to control the activity of CDK9. One way to inactivate CDK9 is by incorporation into the 7SK snRNP [25]. Once it is released from the complex, it activates host cell transcription by promotor-proximal phosphorylation events, which stimulate RNA Pol II pause release [25]. As CDK9 is an important transcription factor [26], its incorporation into the 7SK snRNP complex and its release has to be tightly regulated. One factor activating the release of CDK9 is the DEAD box RNA helicase DDX21 [22], but additional factors have also been identified [25]. Consequently, we analyzed whether AnkG also interacts with the 7SK snRNP. As shown in Fig 3D, NLS-AnkG, but also the non-anti-apoptotic part AnkG_70-338_ binds to GFP-LARP7 and, possibly indirectly, to endogenous CDK9. Importantly, GFP does not bind to either the two AnkG variants or CDK9. These results suggest that NLS-AnkG as well as AnkG_70-338_ interact with the 7SK snRNP. As AnkG_70-338_ is unable to bind to DDX21 (Fig 2B), these data hint to the possibility that the binding of AnkG to the 7SK snRNP is independent of binding to DDX21.

### Binding to DDX21 and/or the 7SK snRNP is essential for the anti-apoptotic activity of AnkG

**Fig 4:**
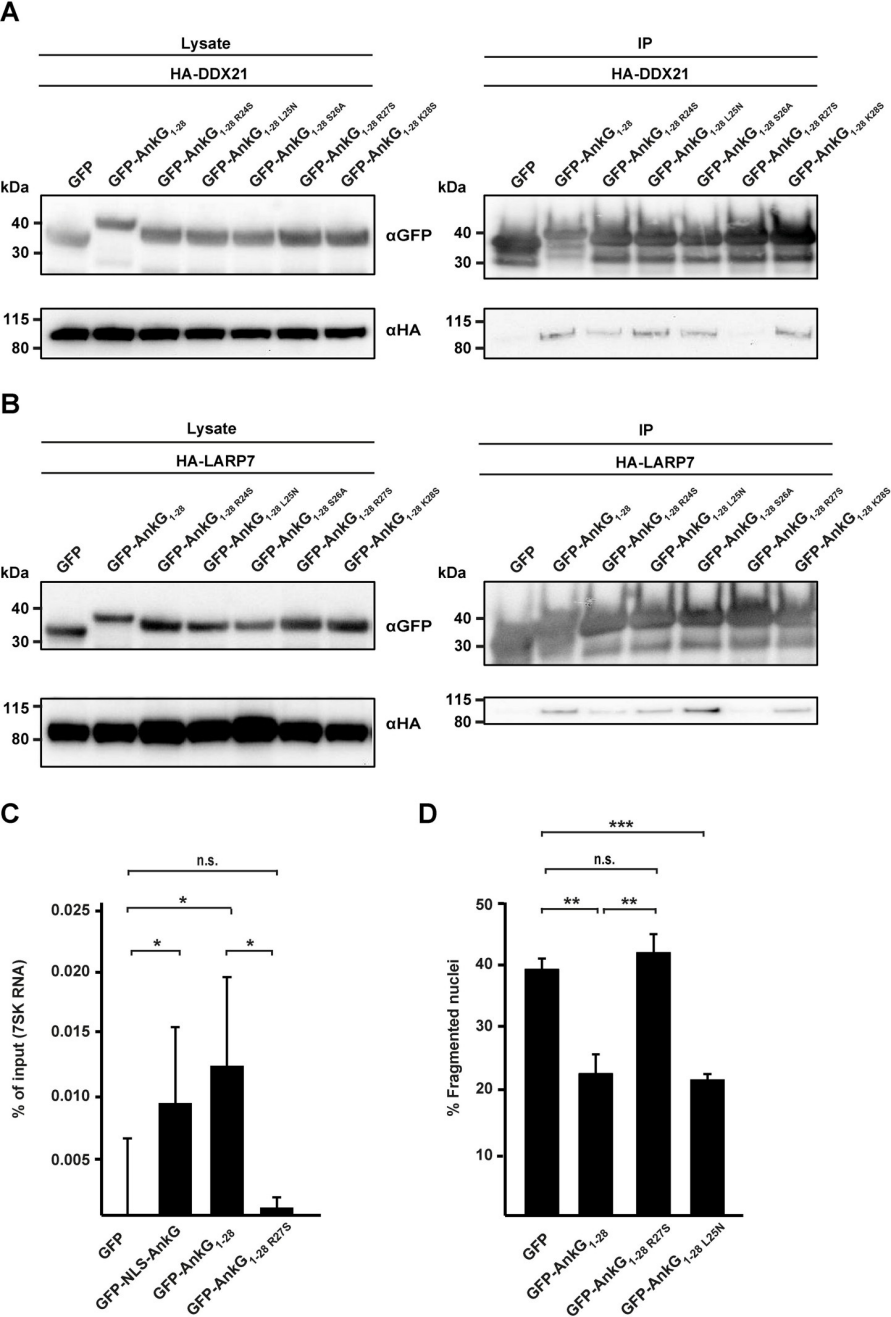
Point mutation at amino acid 27 of AnkG disrupts the AnkG-DDX21 and the AnkG-7SK snRNP interaction and abolishes the anti-apoptotic activity. (A) GFP and GFP-tagged AnkG_1-28_-mutations were transiently co-expressed with HA-DDX21 in HEK293T cells. Proteins were precipitated using GFP-trap. Western blot analysis was used to detect AnkG_1-28_ mutations (anti-GFP) and HA-DDX21 (anti-HA) in the lysates (Pre-IP) and in the precipitates (IP). One out of three independent experiments with similar results is shown. (B) GFP and GFP-tagged AnkG_1-28_-mutations were transiently co-expressed with HA-LARP7 in HEK293T cells. Proteins were precipitated using GFP-trap. Western blot analysis was used to detect AnkG_1-28_ mutations (anti-GFP) and HA-LARP7 (anti-HA) in the lysates and in the precipitates (IP). A representative blot is depicted from three independent experiments with similar results. (C) HEK293T cells were transfected with GFP or GFP-tagged AnkG-variants. RNA-immunoprecipitation was performed using an anti-GFP antibody. The 7SK RNA was reverse transcribed and a qRT-PCR was performed using specific primers. Data presented as percentage of input bound by GFP or GFP-tagged AnkG variants (n = 5). Error bars indicate ± SD. * *p*< 0.05, n. s. = not significant. (D) HeLa cells were transiently transfected with plasmids encoding GFP or GFP-tagged AnkG_1-28_-mutations. Cells were treated with 0.1 μM staurosporine for 4 h, fixed and the DNA was stained with DAPI. The morphology of the nuclei of 100 GFP-expressing cells were scored in three independent experiments. Error bars indicate ± SD. * *p*<0.05, ** *p*< 0.01, n. s. = not significant.

To determine if binding of AnkG to DDX21 is essential for the anti-apoptotic activity we generated different AnkG_1-28_ mutants. In particular, we mutated the amino acid 24 from arginine (R) to serine (S), amino acid 25 from leucine (L) to asparagine (N), amino acid 26 from serine (S) to alanine (A), amino acid 27 from arginine (R) to serine (S) and amino acid 28 from lysine (L) to serine (S). Thus, we changed the charge, steric configuration, or hydrophobicity of the side chain in the respective amino acids. Only one of these mutants, AnkG_1-28 R27S_, was unable to co-precipitate with DDX21 in transfected HEK293T cells (Fig 4A). To characterize this interaction in more detail we generated additional mutants. Arginine (R) at amino acid 27 was substituted with glutamic acid (E), lysine (K), or glutamine (Q). While AnkG_1-28 R27K_ bound to DDX21, this was not the case for the two other additional mutants (S2A Fig). We concluded from this experiment that salt bridge(s) might mediate the binding of AnkG to DDX21.

With the AnkG_1-28 R27S_ mutant, we were now able to determine whether the binding of AnkG to DDX21 influences binding to the 7SK snRNP, and whether the anti-apoptotic activity of AnkG depends on binding to DDX21. First, we analyzed the binding capacity of AnkG_1-28_, which indeed co-precipitated HA-tagged LARP7 (Fig 4B) and the 7SK RNA (Fig 4C). In contrast, AnkG_1-28 R27S_ was unable to co-precipitate HA-tagged LARP7 (Fig 4B) and the 7SK RNA (Fig 4C). These data suggest that the binding of AnkG to the 7SK snRNP depends on binding to DDX21 or vice versa. However, it might also be possible that the binding region within AnkG for both host cell binding partners overlap. Importantly, cells expressing GFP-AnkG_1-28 R27S_ were not protected from staurosporine-induced apoptosis in contrast to cells expressing either GFP-AnkG_1-28_, GFP-AnkG_1-28 L25N_ (Fig 4D) or GFP-AnkG_1-28 R27K_ (S2B Fig). Thus, if AnkG is unable to bind to the 7SK snRNP and/or DDX21, it loses its anti-apoptotic activity.

### The DDX21-AnkG interaction is independent from the 7SK snRNP complex

**Fig 5:**
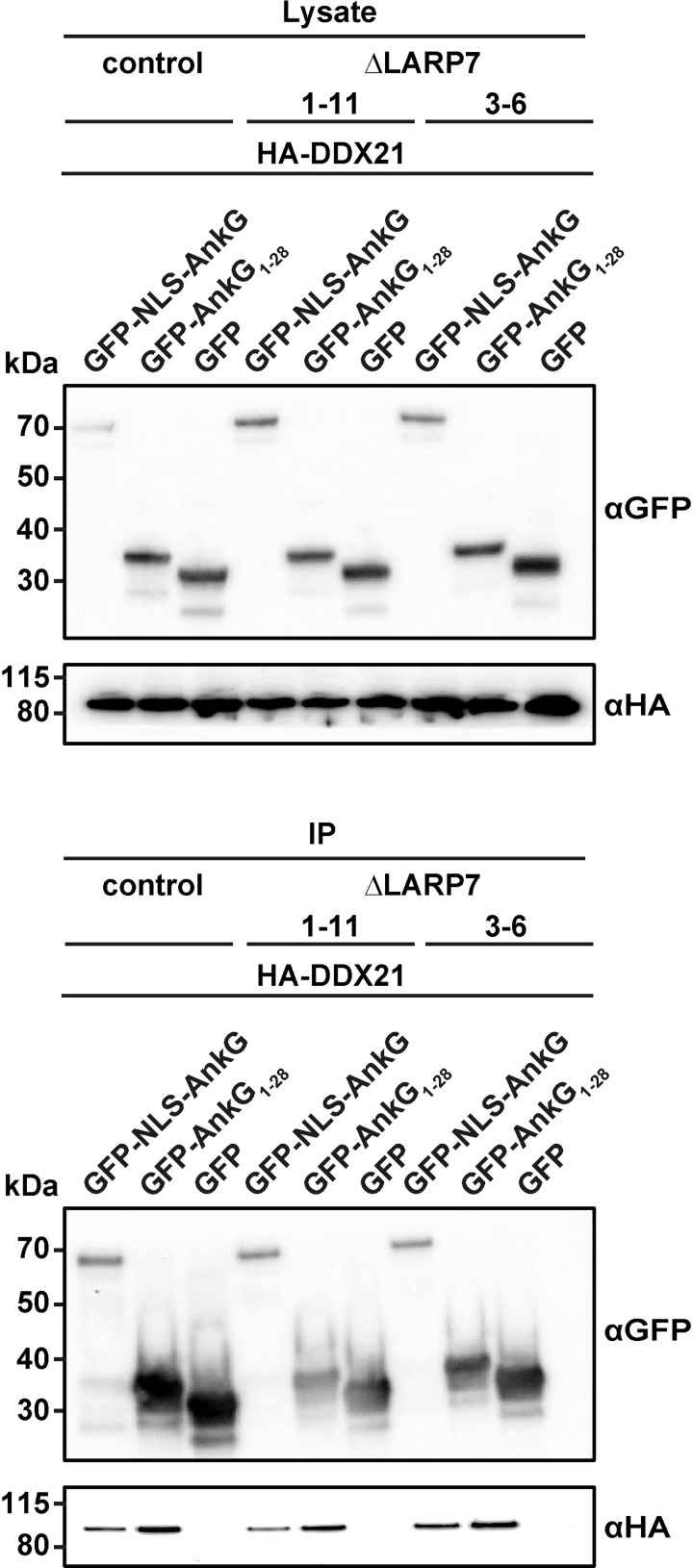
Binding of DDX21 to AnkG is not mediated by LARP7 or 7SK. Two HEK293T mutant cell lines lacking the 7SK RNA (ΔLARP7 1–11 and ΔLARP7 3–6) and a respective control were co-transfected with HA-tagged DDX21 and GFP, GFP-NLS-AnkG or GFP-AnkG_1-28_. Proteins were precipitated using GFP-trap. Western blot analysis was used to detect GFP or GFP-tagged AnkG variants (anti-GFP) and HA-DDX21 (anti-HA). Representative immunoblots out of three independent experiments with similar results are shown.

To elicit whether the co-precipitation of AnkG to DDX21 depends on interaction of AnkG with the 7SK snRNP, we utilized ΔLARP7 HEK293T cells for co-immunoprecipitation experiments [27]. As LARP7 is an important scaffolding protein of the 7SK snRNP complex, its deletion triggers degradation of the complex [28,29]. HA-tagged DDX21 co-precipitated with GFP-NLS-AnkG and GFP-AnkG_1-28_, but not with GFP in control cells and in ΔLARP7 cells, demonstrating that the interaction of AnkG with DDX21 is independent of the 7SK snRNP complex (Fig 5).

### DDX21 is essential for the anti-apoptotic activity of AnkG

**Fig 6:**
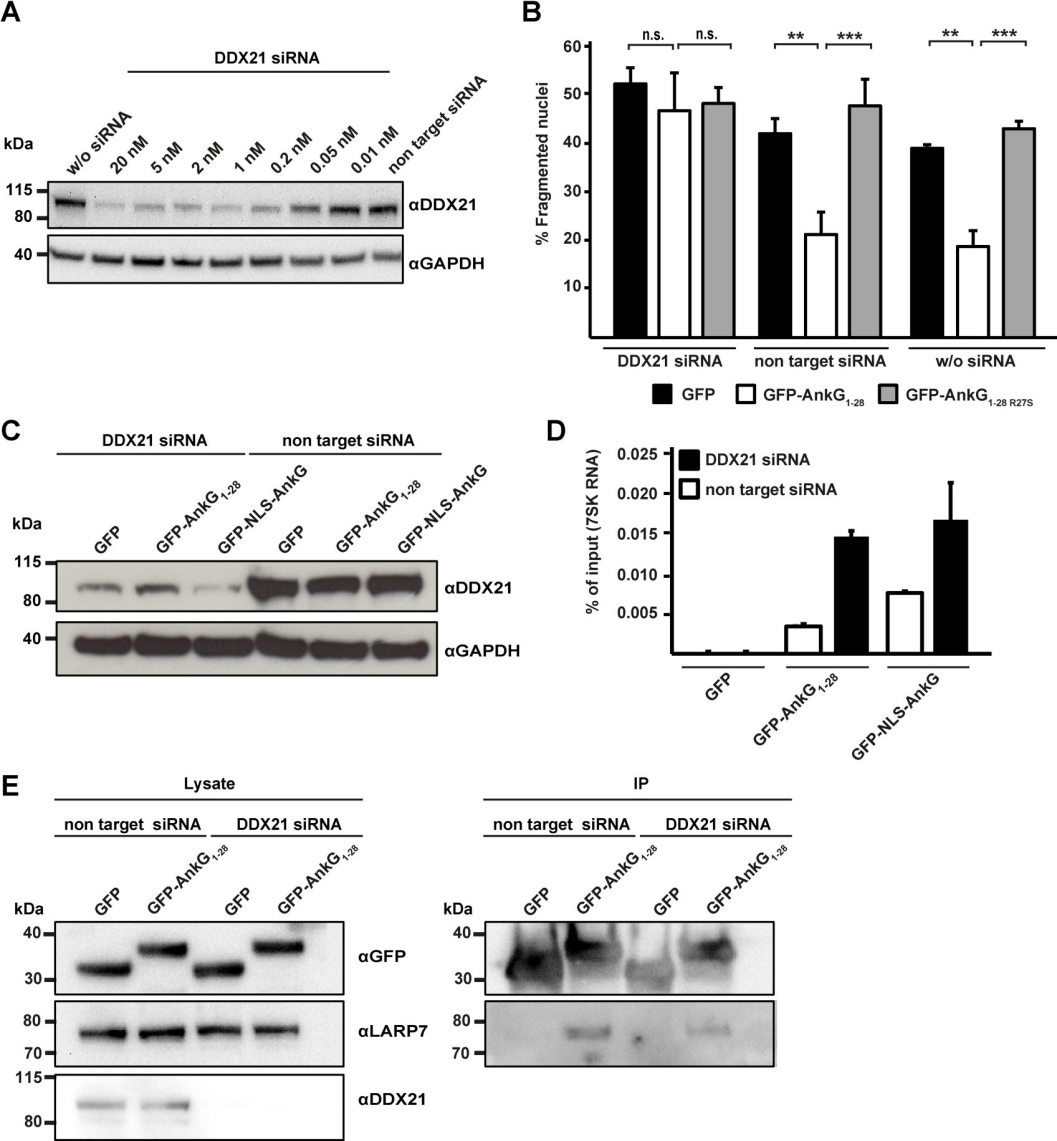
DDX21 is essential for AnkG-mediated apoptosis inhibition, but dispensable for AnkG-7SK snRNP binding. (A) HeLa cells were either not treated, treated with non-target siRNA or with a range of DDX21 siRNA concentrations for 48 h. Cell lysates were analyzed by immunoblot analysis with an anti-DDX21 and an anti-GAPDH antibody as loading control. A representative immunoblot out of two independent experiments with similar results is shown. (B) HeLa cells either not treated, treated with non-target siRNA or with 2 nM DDX21 siRNA for 24 h, were transfected with plasmids encoding GFP, GFP-AnkG_1-28_ and GFP-AnkG_1-28 R27S_. At 20 h post-transfection, cells were treated with 0.1 μM staurosporine for 4 h, fixed and the DNA was stained with DAPI. The morphology of the nuclei of 100 GFP-expressing cells were scored in four independent experiments. Error bars indicate ± SD. ** *p*< 0.01, *** *p*< 0.001, n. s. = not significant. (C—E) HEK293T cells either treated with non-target siRNA or with 10 nM DDX21 siRNA for 24 h were transfected with plasmids encoding GFP, GFP-AnkG_1-28_ and GFP-NLS-AnkG. (C) At 24 h post-transfection DDX21 knock-down was analyzed by immunoblot analysis using an anti-DDX21 antibody and an anti-GAPDH antibody as loading control. (D) RNA-immunoprecipitation was performed using an anti-GFP antibody. The 7SK RNA was reverse transcribed and a qRT-PCR was performed using specific primers. Data presented as percentage of input bound by GFP or GFP-tagged AnkG variants from one representative experiment out of three independent experiments. (E) At 24 h post-transfection proteins were precipitated using GFP-trap. Western blot analysis was used to detect GFP and GFP-AnkG_1-28_ (anti-GFP), endogenous LARP7 (anti-LAPR7) and endogenous DDX21 to confirm knock-down. Representative immunoblots out of three independent experiments with similar results are shown.

We wanted to clarify whether the interaction of AnkG with DDX21 is essential for binding to the 7SK snRNP and/or its anti-apoptotic activity. To this end, we transfected HeLa cells with DDX21 siRNA, which reduced DDX21 protein level in a dose-dependent manner (Fig 6A). The expression of GFP-AnkG_1-28_, but not of GFP or GFP-AnkG_1-28 R27S_ protected the cells from staurosporine-induced apoptosis in cells either mock treated or treated with non-targeting siRNA (Fig 6B). Importantly, in cells treated with DDX21 siRNA the expression of GFP-AnkG_1-28_ did not protect the cells from staurosporine-induced apoptosis (Fig 6B). We concluded from these data, that AnkG requires DDX21 to facilitate its anti-apoptotic activity. Next, we analyzed whether AnkG requires DDX21 for the co-isolation of the 7SK RNA. Therefore, we transfected HEK293T cells with either DDX21 or non-targeting siRNA and co-transfected the cells with GFP, GFP-AnkG_1-28_ or GFP-NLS-AnkG. DDX21 knock-down efficiency was confirmed by immunoblot (Fig 6C). The 7SK RNA bound to GFP-NLS-AnkG and GFP-AnkG_1-28_ independent of the presence or absence of DDX21 (Fig 6D). In addition, endogenous LARP7 co-precipitated with GFP-AnkG_1-28_ independently of the presence of DDX21 (Fig 6E).

Thus, the binding of AnkG to the 7SK snRNP is independent of the binding to DDX21. From these data, we hypothesize that AnkG might influence DDX21 activity, which results in alteration of host cell transcription controlled by the 7SK snRNP complex.

### AnkG expression influences host cell transcription

**Fig 7:**
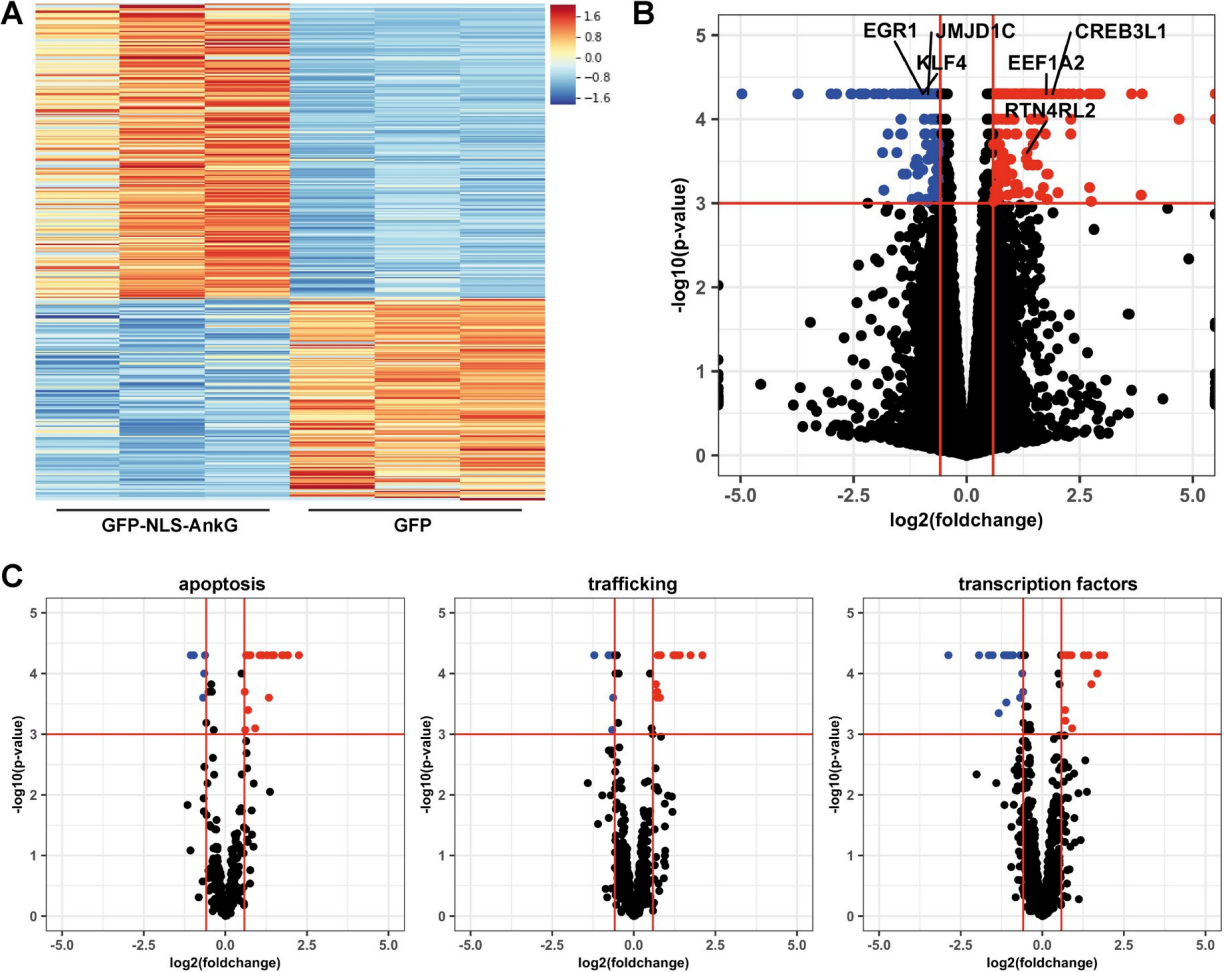
Expression of AnkG alters host cell transcription. HEK293T cells were transfected with plasmids encoding GFP or GFP-NLS-AnkG. GFP-positive cells were sorted by GFP signal and total RNA was isolated and sequenced. Gene expression of cells transfected with NLS-AnkG was compared to GFP expressing control cells in three independent experiments. (A) Heat map of 361 differentially expressed genes (defined as fold change > 1.5 with p-value of 0.001) in GFP-NLS-AnkG expressing cells relative to GFP expressing control cells. (B) Shown is the volcano plot of differential gene expression. Significant differential expressed genes are indicated in red (upregulated) and blue (downregulated). Genes validated by RT-PCR are named. (C) Volcano plot of genes associated to either apoptosis, trafficking or transcription. The significant downregulated genes (blue) and upregulated genes (red) are shown.

To determine whether AnkG modulates host cell transcriptional activity, we ectopically expressed either GFP-NLS-AnkG or GFP as a control in HEK293T cells, FACS sorted GFP-positive cells and isolated RNA for RNAseq analysis. To examine gene expression pattern of GFP-NLS-AnkG expressing cells with GFP expressing cells we constructed a heat map. The criteria for the listed genes were >1.5 log fold change and a minimal FPKM value of 1 (Fig 7A). In comparison to cells expressing GFP, 361 significant differentially regulated genes were identified in cells expressing GFP-NLS-AnkG, with 215 genes upregulated and 146 genes downregulated (Fig 7B, geo accession number: GSE185428, S2 Table). The majority of genes deregulated by expression of GFP-NLS-AnkG can be grouped into three different gene ontology pathways: i) apoptosis, ii) trafficking and iii) transcription factors (Fig 7C). The 10 most up- and down-regulated genes are shown in Tables 2 and 3. These genes encode RNAs, chaperones, transcription factors or proteins involved in calcium mediated processes, mitochondrial function, pH regulation or trafficking. Importantly, in most cases genes coding for pro-apoptotic proteins were down-regulated, while those encoding anti-apoptotic proteins were up-regulated by AnkG-expression. For a selection of the affected genes (Fig 7B), the RNAseq results were confirmed by qRT-PCR (S3 Fig).

**Table 2:** Top 10 differentially up-regulated genes.

Gene label	Gene product	Log	p value	Biological processes
RNU6ATAC	RNA, U6atac small nuclear	Inf	0,0001	Spliceosomal tri-snRNP complex assembly mRNA 5´-splice site recognition
AC008764.4	Uncharacterized protein	Inf	0,0001	unknown
AL356432.3	TBL1XR1 pseudogene	4,7	0,0001	unknown
LINC00674	Long Intergenic Non-protein coding RNA 674	3,9	0,0001	unknown
AP000769.1	Uncharacterized protein	3,9	0,0004	unknown
ZNF467	Zinc finger protein 467	3,6	0,0001	Transcription factor
SLC9C2	Sodium/hydrogen exchanger 11	2,9	0,0001	Involved in pH regulation
ATP2A3	ER calcium ATPase 3	2,9	0,0001	Transport of calcium ions
CPNE4	Copine-4	2,8	0,0001	May play a role in calcium-mediated processes
NECTIN4	Nectin-4	2,8	0,0001	Involved in cell adhesion

**Table 3:** Top 10 differentially down-regulated genes.

Gene label	Gene product	Log	p value	Biological processes
COX6B2	Cytochrome c oxidase subunit 6B2	-5,0	0,0001	Drives oxidative phosphorylation
SNX1	Sorting nexin-1	-3,7	0,0001	Intracellular trafficking
HSPA6	Heat shock 70 kDa protein 6	-3,0	0,0001	Molecular chaperone involved in protein quality control
INHBA	Inhibin beta A chain	-2,9	0,0001	Inhibition of the secretion of follitropin
AKNA	Microtubule organization protein	-2,6	0,0001	Microtubule organizer transcription factor
IMMP1L	Mitochondrial inner membrane protease subunit 1	-2,5	0,0001	Protein targeting to mitochondria processes DIABLO
HSPA1B	Heat shock 70 kDa protein 1B	-2,4	0,0001	Molecular chaperone involved in protein quality control
LIPE-AS1	LIPE antisense RNA1	-2,3	0,0001	Affiliates with the lncRNA class
MT-RNR1	Mitochondrial-derived peptide MOTS-c	-2,2	0,0001	Regulates insulin homeostasis inhibits the folate cycle
AC005776.2	pseudogene	-2,0	0,0001	unknown

Taken together, our results suggest that AnkG influences transcription, and in particular impacts apoptosis-regulating genes towards an anti-apoptotic phenotype. These data correlate with the pro-survival activity of AnkG [14–17].

### AnkG´s anti-apoptotic activity depends on CDK9

**Fig 8:**
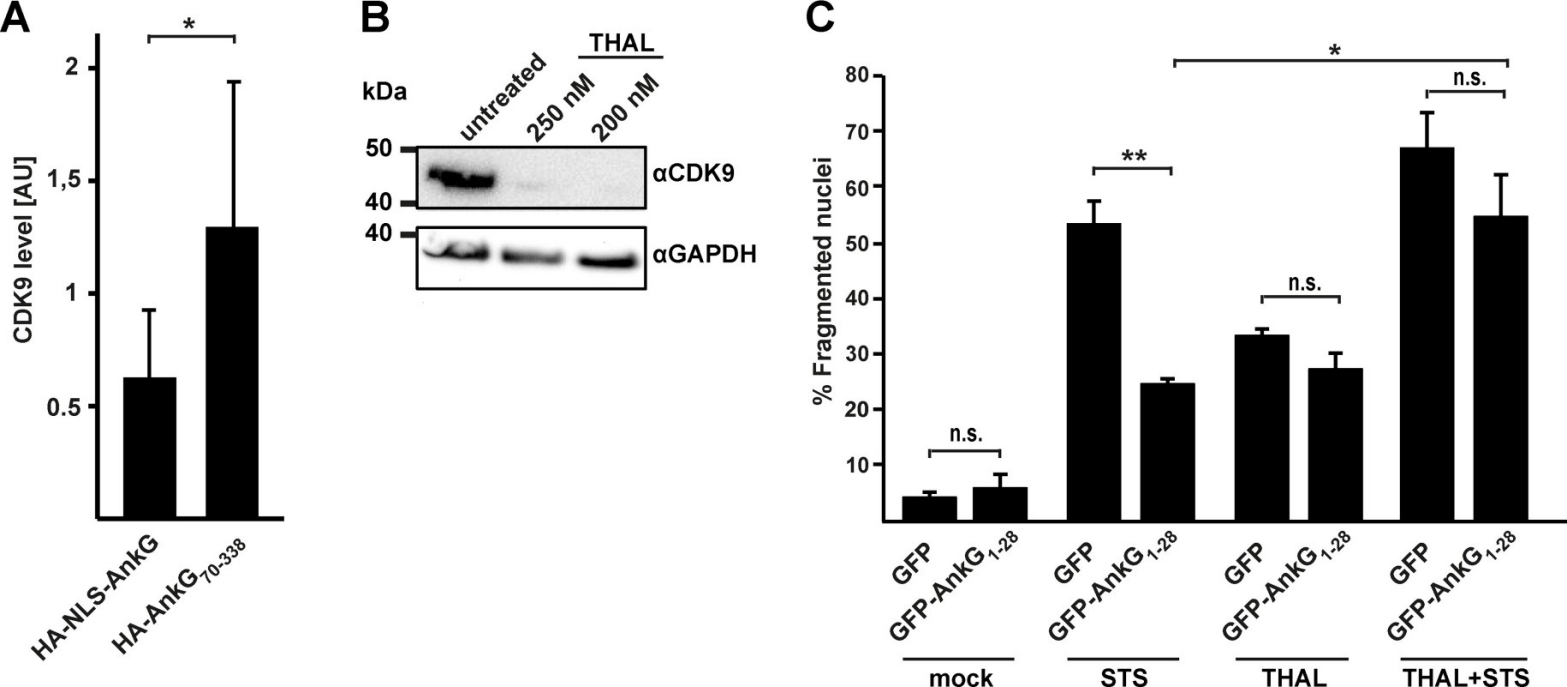
The anti-apoptotic activity of AnkG depends on the release of CDK9 from the 7SK snRNP. (A) HEK293T cells were co-transfected with HA-tagged AnkG-variants and GFP-LARP7. Proteins were precipitated using GFP-trap. Western blot analysis was used to detect AnkG variants (anti-HA), GFP-LARP7 (anti-GFP) and endogenous CDK9 (anti-CDK9) in the precipitates (IP). Densitometry analysis of CDK9 level in relation to the LARP7 level was performed from six independent experiments. Error bars indicate ± SD. * *p*< 0.05. (B) HeLa cells were either left untreated or were treated with indicated concentrations of the CDK9 inhibitor THAL SNS 032 (THAL) for 4 hours. Cell lysates were subjected to western blot analysis using antibodies directed against CDK9 and GAPDH as loading control. (C) GFP and GFP-AnkG_1-28_ were transiently expressed in HeLa cells. The cells were either left untreated or were treated with 0.1 μM staurosporine (STS) in combination with or without 200 nM of THAL for 4 hours. The cells were fixed and the nuclei were stained with DAPI. The nuclear morphology of 100 transfected cells was determined by epifluorescence microscopy. Shown is the mean of three independent experiments. Error bars indicate ± SD. ** *p*<0.01, n. s. = not significant.

We hypothesized that the anti-apoptotic activity of AnkG is linked to its ability to influence transcription via modulation of the 7SK snRNP complex. This complex keeps P-TEFb in an inactive state. Once P-TEFb is released it phosphorylates a number of substrates at promotors, which ablate Pol II pausing [21]. As pausing of Pol II is a major checkpoint in the transcription cycle [30], release of P-TEFb from the 7SK snRNP results in transcription activity. In addition, released CDK9 acts on several host cell pathways and this correlates with increased host cell viability and reduced apoptosis [22,31]. To determine whether AnkG mediates release of CDK9, the P-TEFb kinase, we performed a CDK9 release assay. HEK293T cells expressing either HA-NLS-AnkG or HA-AnkG_70-338_ and GFP-LARP7 were analyzed via GFP-trap to precipitate all proteins interacting with GFP-LARP7. Importantly, both AnkG variants bound LARP7 (Fig 3D). Comparing the amount of endogenous CDK9 with the level of LARP7 protein, we can indirectly evaluate the release of CDK9 from the complex. As shown in Fig 8A, the amount of CDK9 bound to LARP7 is significantly higher in cells expressing the non-anti-apoptotic AnkG_70-338_ than in cells expressing NLS-AnkG. This data suggests that AnkG might stimulate the release of CDK9 from the complex. If AnkG´s anti-apoptotic activity depends on its ability to release CDK9 from the 7SK snRNP in a DDX21-dependent manner, the degradation of CDK9 should abolish AnkG´s anti-apoptotic action. Pharmacological inhibition of CDK9 is challenging as some inhibitors target several CDKs. The small molecule THAL-SNS-032 (THAL) however has been proven to induce potent and selective degradation of CDK9 [32]. After confirming efficient elimination of CDK9 by THAL (Fig 8B), we analyzed whether the presence or absence of CDK9 influences the activity of GFP-AnkG_1-28_. While GFP-AnkG_1-28_ expression protected the cells from staurosporine-induced apoptosis, as shown before [17], it did not protect from THAL-induced apoptosis (Fig 8C). Treatment with both, staurosporine and THAL, increased the rate of apoptotic cells. Importantly, the percentage of apoptotic cells did not differ between cells expressing GFP or GFP-AnkG_1-28_ under these conditions. This indicates that AnkG is unable to prevent apoptosis in the absence of CDK9 and supports our assumption that AnkG modulates host cell viability by DDX21-dependent activation of 7SK snRNP-controlled transcription.

### AnkG is important for *C*. *burnetii*-mediated apoptosis inhibition

**Fig 9:**
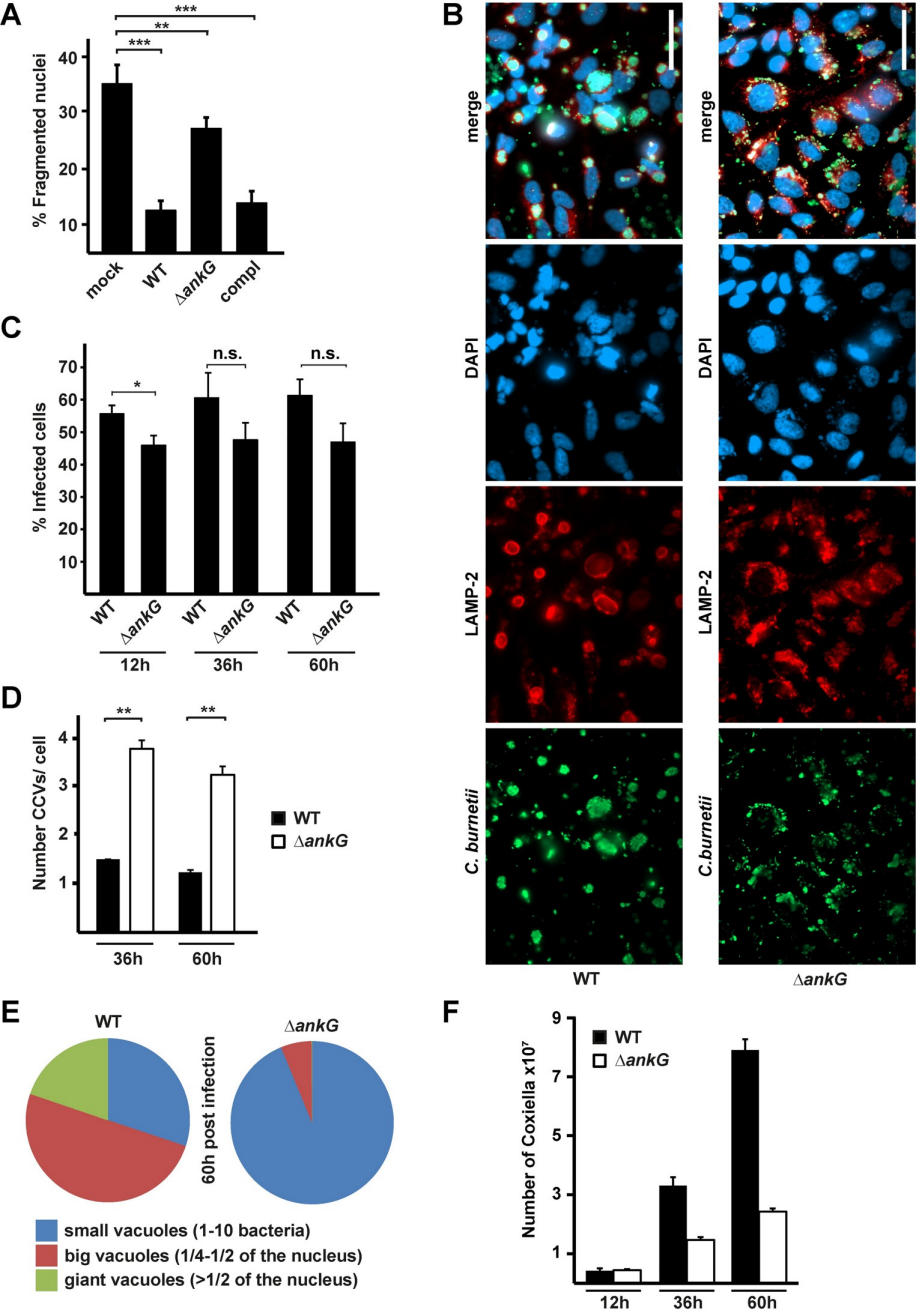
AnkG is essential for *C*. *burnetii*-mediated apoptosis-inhibition and CCV biogenesis. (A) HeLa cells either not infected (mock), infected with wild-type *C*. *burnetii* (WT), *C*. *burnetii* Δ*ankG* (Δ*ankG*) or *C*. *burnetii* Δ*ankG*::AnkG (compl) were treated with 0.1 μM staurosporine for 4 h, fixed and the DNA was stained with DAPI. The morphology of the nuclei of 100 GFP-expressing cells were scored in three independent experiments. Error bars indicate ± SD. ** *p*< 0.01, *** *p*< 0.001, n. s. = not significant. (B—F) HeLa cells were infected with *C*. *burnetii* wild-type (WT) and an *ankG* deletion strain (Δ*ankG*). After 48 hours the cells were fixed and stained with antibodies specific for *C*. *burnetii* (green), DAPI (blue) and an anti-LAMP-2 antibody (red). (B) Representative images of three independent experiments with similar results are shown. Scale bar 50μm. (C) The infection rate was determined at 12, 36 and 60 hours post-infection of 100 cells each using an epifluorescence microscope. Shown is the mean of three independent experiments. Error bars indicate ± SD. * *p*< 0.05, n. s. = not significant. (D—E) Representative images of three independent experiments were used to determine the (D) vacuole number at 36 and 60 hours post-infection and (E) vacuole size in 100 infected cells each at 60 hours post-infection. Shown is the mean of three independent experiments. Error bars indicate ± SD. ** *p*< 0.01. (F) *C*. *burnetii* counts of either wild-type (WT) or *C*. *burnetii* Δ*ankG* (Δ*ankG*) were determined at indicated time points via counting of colony forming units (CFU). Shown is a representative experiment out of four experiments with similar results, performed with technical triplicates.

While it has been demonstrated that AnkG possesses anti-apoptotic activity [14–17], AnkG-mediated anti-apoptotic activity has not been shown to be important during *C*. *burnetii* infection. Consequently, we generated an *ankG* deletion strain (Δ*ankG*) by homologous recombination and a corresponding complemented strain (Δ*ankG*::AnkG) using a Tn7 construct to introduce *ankG* into the chromosome under the control of an insulated bacterial promotor [17,33]. We infected HeLa cells with Δ*ankG*, Δ*ankG*::AnkG or with wild-type *C*. *burnetii*, induced intrinsic apoptosis by staurosporine treatment and analyzed apoptosis-induction in infected cells. While *C*. *burnetii*-infection protected the cells from apoptosis (Fig 9A) [34,35], the *ankG* deletion mutant was significantly impaired in its anti-apoptotic activity. Nevertheless, this mutant still has residual anti-apoptotic activity, which could be expected, as *C*. *burnetii* harbors multiple anti-apoptotic T4SS effector proteins [36,37]. As the complemented strain showed restored wild-type anti-apoptotic activity, we assumed that the anti-apoptotic defect of the Δ*ankG* mutant was mediated by the lack of the T4SS effector protein AnkG. To exclude that this phenotype was rather due to a reduced ability to infect host cells, we analyzed infection and replication of the Δ*ankG* mutant in more detail.

### AnkG is essential for establishing the replicative *C*. *burnetii*-containing vacuole (CCV)

While wild-type *C*. *burnetii* generally established a single large LAMP-2 positive CCV per cell at 60 h post-infection, the Δ*ankG* mutant infection produced multiple smaller LAMP-2 positive CCVs per cell (Fig 9B). Importantly, the infection rate was in a similar range between the mutant and the wild-type at 12, 36 and 60 h post-infection (Fig 9C), suggesting that AnkG is dispensable for internalization of *C*. *burnetii*. In contrast, AnkG seems to be important for homotypic fusion of the CCVs. It has been reported that after infection, *C*. *burnetii* establishes multiple small CCVs, which fuse over time to generate one large CCV per cell [38]. While we observed that cells infected with the wild-type generally established a single large CCV per cell at 60 h post-infection, the Δ*ankG* mutant established three to four small CCVs per cell at this time point (Fig 9D and 9E). We determined the colony-forming units (CFUs) from cells infected with the wild-type and the Δ*ankG* mutant, to learn whether the defect in homotypic fusion of the CCVs has an impact on efficient replication. As shown in Fig 9F the Δ*ankG* mutant infected cells as efficient as wild-type bacteria, but was significantly inhibited in its ability to replicate intracellularly. Thus, AnkG is important for efficient replication. To analyze whether this depends on its anti-apoptotic activity, we characterized infection rates and CCV characteristics of the Δ*ankG* mutant and the wild-type bacteria in HeLa cells either overexpressing the anti-apoptotic Bcl-x_L_ or lacking the pro-apoptotic Bax/Bak proteins. Deletion of Bax/Bak or overexpression of Bcl-x_L_ blocks intrinsic apoptosis [39,40]. As neither the infection-rate, nor the characteristics of the CCVs was altered in these two different cell lines (S4 Fig), we concluded that the role of AnkG for intracellular replication and CCV formation is not linked to its anti-apoptotic activity. Further research is necessary to clarify which other pathway(s) or factor(s) deregulated by AnkG are involved in this phenotype.

### AnkG also influences host cell transcription during infection

**Fig 10:**
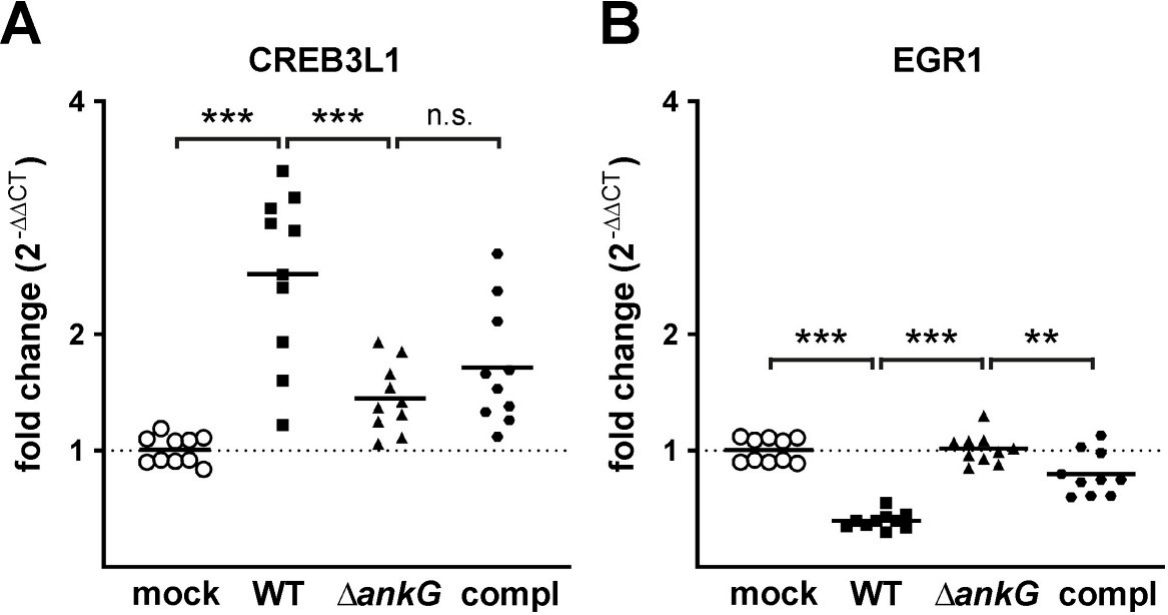
AnkG induced transcriptional reprogramming during infection. HeLa cells were either uninfected (mock) or infected for 72 hours with wild-type *C*. *burnetii* (WT), *C*. *burnetii* Δ*ankG* (Δ*ankG*) or *C*. *burnetii* Δ*ankG*::AnkG (compl). Total RNA was isolated and reverse transcribed in cDNA using SuperScript II reverse transcriptase according to the manufacturer’s protocol. A qRT-PCR was performed with primers amplifying fragments of the indicated genes. The ΔΔCt values were calculated for the fold difference of expression in uninfected versus infected cells.

As the Δ*ankG* mutant has reduced anti-apoptotic activity in comparison to wild-type *C*. *burnetii* (Fig 9A), we wanted to know whether this could be caused by the lack of AnkG-mediated alteration of host cell transcription. Thus, we infected HeLa cells with wild-type *C*. *burnetii*, Δ*ankG* or Δ*ankG*::AnkG for 3 days, isolated RNA and performed qRT-PCR to determine the expression level of EGR1 and CREB3L1 compared to uninfected cells. We chose these two genes as EGR1 was up-regulated and CREB3L1 was down-regulated by AnkG ectopic expression (S3 Fig). Infection of HeLa cells with wild-type *C*. *burnetii* resulted in up-regulation of CREB3L1, which was not seen when the cells were infected with the Δ*ankG* mutant (Fig 10A). In addition, infection with wild-type *C*. *burnetii*, but not with the Δ*ankG* mutant resulted in down-regulation of EGR1 (Fig 10B). In both cases, the complemented strain only partially complemented the phenotype of the mutant. The underlying reason for this is currently unknown and has to be analyzed. Nevertheless, these data suggest that also the translocated effector protein AnkG influences host cell transcription similar to the activity of ectopic expressed AnkG.

### AnkG is important for *C*. *burnetii*-mediated apoptosis inhibition and establishment of the CCV in infected THP-1 cells

**Fig 11:**
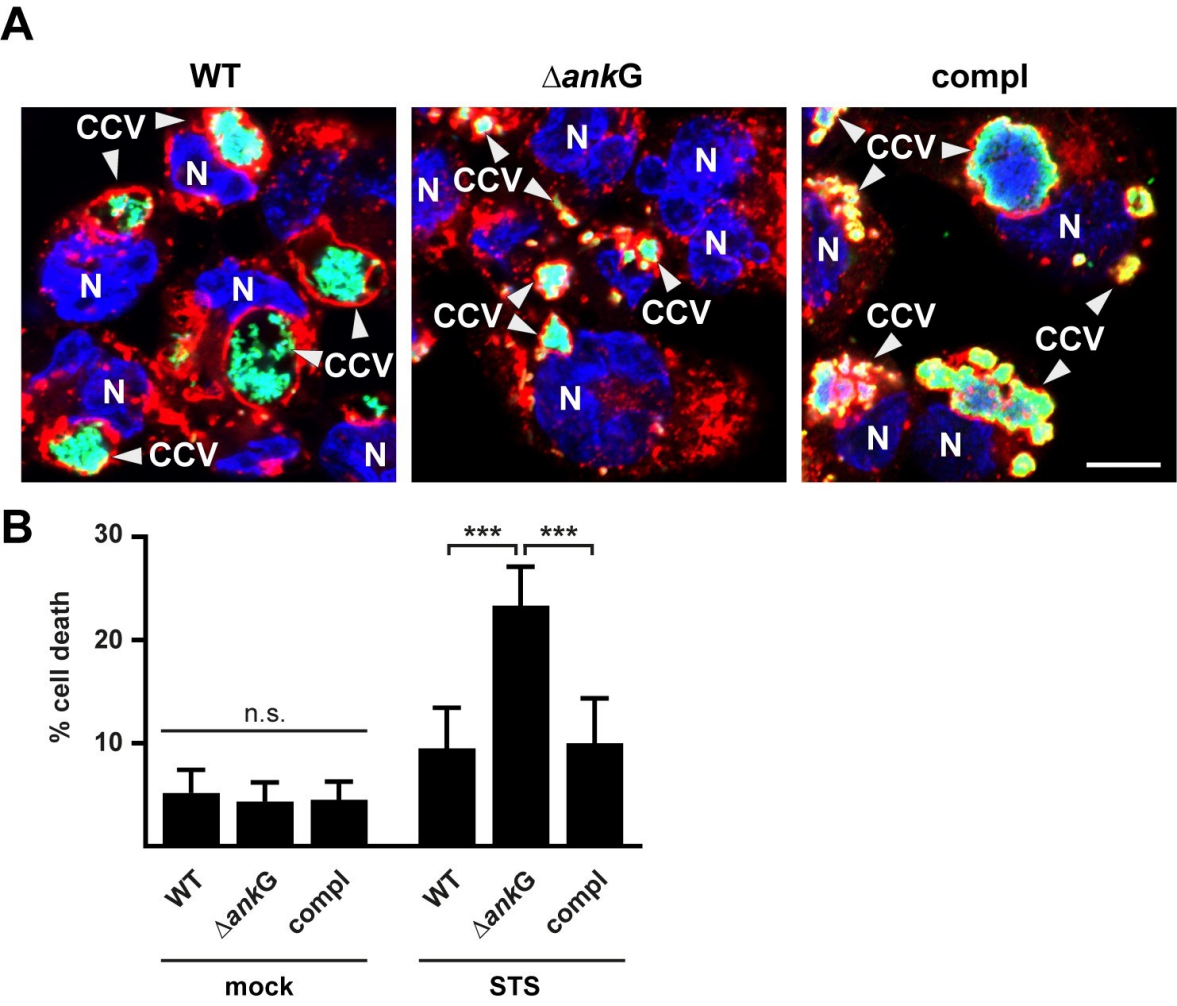
AnkG is important for anti-apoptotic activity and CCV formation during infection in THP-1 cells. (A-B) Differentiated THP-1 cells were either infected with wild-type *C*. *burnetii* (WT), *C*. *burnetii* Δ*ankG* (Δ*ankG*) or *C*. *burnetii* Δ*ankG*::AnkG (compl) for 72 h. (A) The cells were fixed and stained with antibodies against LAMP-2 (red) and *C*. *burnetii* (green). The host cell nucleus and bacterial DNA was stained with DAPI (blue). Representative immunofluorescence images are shown. Scale bar 10 μm. (B) Cells were treated with 0.5 μM staurosporine (STS) for 4 h. The morphology of the nuclei of 100 infected cells was scored in two independent experiments performed in duplicates. Error bars indicate ± SD. *** *p*< 0.001 n. s. = not significant.

The results presented so far were obtained using HEK293T and HeLa cells. However, the primary target cells of *C*. *burnetii* are macrophages. Therefore, we infected differentiated THP-1 cells with wild-type *C*. *burnetii*, Δ*ankG* or Δ*ankG*::AnkG, as they have properties of human monocyte-derived macrophages [35], and analyzed the role of AnkG for CCV formation and anti-apoptotic activity. As shown in Fig 11A the deletion of AnkG resulted in smaller CCVs. The Δ*ankG* mutant also induced a higher cell death rate in infected THP-1 cells (Fig 11B), confirming our results. Thus, also in THP-1 cells AnkG is important for the anti-apoptotic activity of *C*. *burnetii* and for the formation of the CCV.

### DDX21 is important for *C*. *burnetii*-mediated anti-apoptotic activity and CCV formation

**Fig 12:**
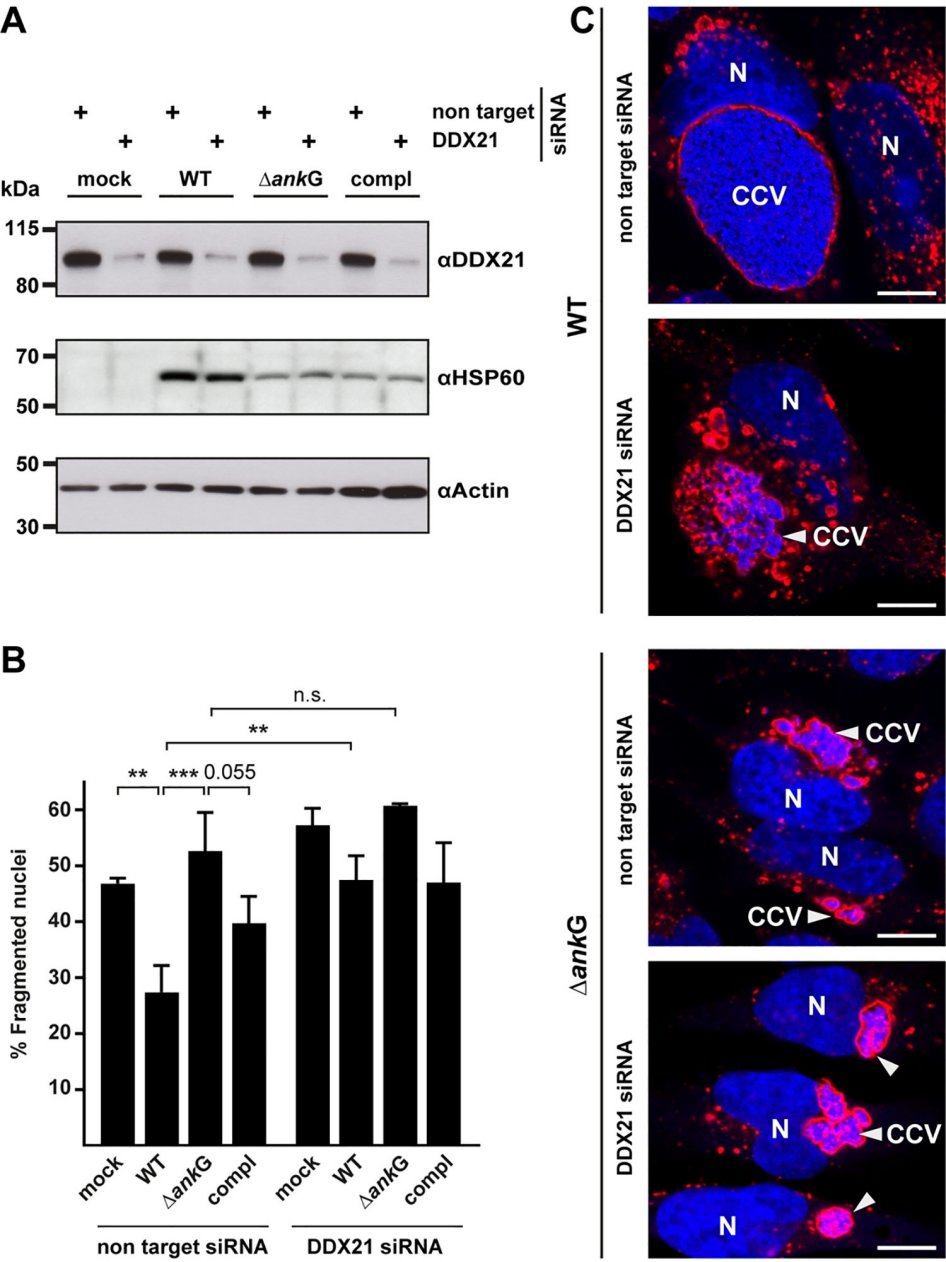
DDX21 is important for anti-apoptotic activity and CCV formation during *C*. *burnetii* infection. (A-C) HeLa cells were either uninfected (mock), infected with wild-type *C*. *burnetii* (WT), *C*. *burnetii* Δ*ankG* (Δ*ankG*) or *C*. *burnetii* Δ*ankG*::AnkG (compl). 24 hours post-infection cells were either treated with non-target siRNA or with DDX21 siRNA for 48 hours. (A) Cell lysates were subjected to western blot analysis using antibodies directed against DDX21, bacterial HSP60 for bacterial loading control and actin as loading control. (B) Cells were treated with 0.1 μM staurosporine for 4 h, fixed and stained with an antibody against LAMP-2. The host cell nucleus and bacterial DNA was stained with DAPI. The morphology of the nuclei of 100 infected cells was scored in three independent experiments. Error bars indicate ± SD. ** *p*< 0.01, *** *p*< 0.001 n. s. = not significant. (C) Representative immunofluorescence images are shown. Scale bar 10 μm.

Our data indicates that the anti-apoptotic activity of AnkG depends on binding to DDX21 (Fig 6B). However, whether DDX21 is also important for anti-apoptotic activity during *C*. *burnetii* infection was unresolved. To answer this question we infected HeLa cells with wild-type *C*. *burnetii*, Δ*ankG* or Δ*ankG*::AnkG for 24 hours. The cells had been either treated with non-targeting siRNA or DDX21 siRNA for 48 hours. DDX21 knock-down efficiency was confirmed by immunoblot (Fig 12A) before apoptosis was induced. In cells treated with DDX21 siRNA infection with wild-type *C*. *burnetii* did not protect the cells from staurosporine-induced apoptosis (Fig 12B). We concluded from these data, that DDX21 is required for the *C*. *burnetii*-mediated anti-apoptotic activity. In a next step, we analyzed the morphology of the CCV in DDX21 knock-down cells. While wild-type *C*. *burnetii* establishes one large CCV per cell treated with non-targeting siRNA, we observed an altered morphology of the CCV in cells incubated with DDX21 siRNA (Fig 12C). DDX21 seems to be important for homotypic fusion, similar to the role of AnkG in CCV biogenesis (Figs 12C and 9D and 9E). However, while the lack of AnkG resulted in reduced replication of *C*. *burnetii* (Figs 9F and 12A), the lack of DDX21 seems not to reduce bacterial replication as demonstrated by comparable bacterial HSP60 levels (Fig 12A).

These data suggest that AnkG acts via DDX21 and the 7SK snRNP complex during infection, but AnkG might also interfere with other host cell factors independently of the DDX21/7SK snRNP complex.

## Discussion

AnkG is injected into the host cell in a T4SS-dependent manner [41]. While a subset of *C*. *burnetii* T4SS effector proteins require the chaperone IcmS for translocation [42], this is not the case for AnkG [43]. Ectopically expressed AnkG associates with the host cell mitochondria and migrates under apoptotic stress conditions into the host cell nucleus [14,16]. During *C*. *burnetii* infection, translocated AnkG exclusively localizes within the host cell nucleus [16]. For its anti-apoptotic activity, the first 28 amino acids are essential [17]. Inhibition of host cell apoptosis is important for this obligate intracellular pathogen, as it allows the completion of the lengthy intracellular replication cycle [44]. The T4SS is essential for inhibiting host cell apoptosis [12], underlining the importance of anti-apoptotic effector proteins in pathogenicity. Other intracellular pathogens also subvert the apoptotic cascade via secreted effector proteins [45], and *C*. *burnetii* harbors several apoptosis-regulating effector proteins [18]. As AnkG has to localize within the host cell nucleus in order to execute its anti-apoptotic activity [14,16], it can be categorized as a nucleomodulin, which are bacterial effector proteins targeting the host cell nucleus [20]. So far, a few *C*. *burnetii* nucleomodulins have been identified: CBU0129, CBU388, CBU393, CBU0781 (AnkG), CBU0794, CBU0937, CBU1217 (NopA), CBU1314, CBU1524 and CBUK1976 [13,16,46–48]. However, as it has been proven challenging to visualize subcellular localization of translocated *C*. *burnetii* T4SS effector proteins, the localization of most of the *C*. *burnetii* nucleomodulins has been only determined when expressed ectopically in mammalian cells. Some of these nucleomodulins have been studied in more detail. CBU0388 was shown to be important for intracellular replication [48], to modulate MAP kinase activity in yeast and to inhibit yeast growth [49]. CBU1524 (CaeA) inhibits intrinsic and extrinsic apoptosis, which depends on the EK repetition motif of CaeA [37,50]. CBU1314 associates with host chromatin and modulates the host transcriptome [51]. CBU1217 (NopA) associates with chromatin, which perturbs nuclear import of transcription factors of the innate immune signaling pathway [46]. Thus, these two T4SS effector proteins, CBU1314 and NopA, target the host cell chromatin to manipulate the host cell. This is a common strategy of nucleomodulins [20]. Several nucleomodulins from pathogenic bacteria directly impact host chromatin structures to influence regulatory host cell processes [19]. One example is AnkA from *Anaplasma phagocytophilum*. AnkA is translocated by the *A*. *phagocytophilum* T4SS into the host cell [52], where it localizes inside the host cell nucleus [53]. It binds AT-rich DNA regions and downregulates the expression of key host defense genes, including *CYBB* [54]. Repression of *CYBB* is mediated by AnkA-dependent recruitment of histone deacetylase-1 (HDAC1) to the promotor region. HDAC1 deacetylates histone H3 and thereby alters binding of RNA Pol II. As a consequence of HDAC1 activity, gene expression is silenced [55]. Other nucleomodulins are histone-modifying enzymes. The *Chlamydia trachomatis* T3SS effector protein NUE exhibit histone methyltransferase activity and thereby influences gene expression [56]. Similarly, the *Legionella pneumophila* T4SS effector protein RomA/LegAS4 is a histone methyltransferase, which represses gene expression and promotes intracellular replication [57,58]. In contrast, our data suggests that AnkG modulates the 7SK snRNP complex to modulate gene expression (Figs 7A and 7B and 7C and 8A and 10A and 10B) and intracellular replication (Fig 9F). This complex is also targeted by viral proteins to influence viral transcription activity [59]. The Tax protein from the human T-lymphotropic virus type 1 (HTLV-1) binds cyclin T1, which dissociates CDK9 from the complex for transcriptional activation of viral genes [60]. Similarly, the HIV Tat protein interacts with cyclin T1 to promote HIV mediated transcription [61]. The viral tegument protein VP16 of the herpes simplex virus 1 (HSV-1) also interacts with P-TEFb to promote transcription of viral genes. Interestingly, HSV-1 harbors another protein, ICP22, which is also able to recruit P-TEFb to viral gene promotors. In contrast to VP16, it represses viral gene transcription [62]. Thus, ICP22 promotes viral latency, which VP16 can overcome, suggesting that modulation of P-TEFb allows the virus the transition between latent and productive infection [59]. Interestingly, the *L*. *pneumophila* T4SS effector proteins AnkH also interacts with the 7SK snRNP to influence host cell transcription [63]. In contrast to our data demonstrating that AnkG modulates the 7SK snRNP via interaction with DDX21 and the 7SK RNA, AnkH was shown to interact with LARP7 [63], the scaffolding protein of the 7SK snRNP complex [64]. The interaction of AnkH with LARP7 seems to impede interaction of LARP7 with other proteins of this complex, which might result in release of CDK9 from the 7SK snRNP complex. Although this was not analyzed in the study, the finding that AnkH alters host cell transcription supports this hypothesis [63]. Thus, the mechanisms of action of the *L*. *pneumophila* T4SS effector protein AnkH and the *C*. *burnetii* T4SS effector AnkG are quite diverse. Although AnkG also interacts with LARP7 (Fig 4B) our data support a model of AnkG action where AnkG-mediated recruitment of DDX21 to the 7SK snRNP seems to be critical for apoptosis inhibition, as it induces the release of CDK9, which is similarly important for apoptosis inhibition (Fig 8).

While AnkH was shown to be important for intracellular replication, the underlying mechanism(s) have not been identified in detail. In contrast, the AnkG deletion strain is compromised in its ability to inhibit apoptosis and to establish a replicative CCV (Fig 9A and 9B and 9F). Thus, AnkG is a nucleomodulin that also influences CCV biogenesis and/or fusion. So far, the *C*. *burnetii* T4SS effector proteins identified to influence CCV morphology are either localized at the CCV membrane, the membrane of endocytic vesicles or in the host cell cytoplasm [48,65–70]. Importantly, the role of AnkG in CCV formation is not linked to its anti-apoptotic activity (S4 Fig), supporting our RNAseq data, where we showed that AnkG influences not only apoptosis, but also trafficking and transcription factors (Fig 7). Thus, the activity of AnkG is important for several pathways essential for *C*. *burnetii* intracellular survival and replication.

**Fig 13:**
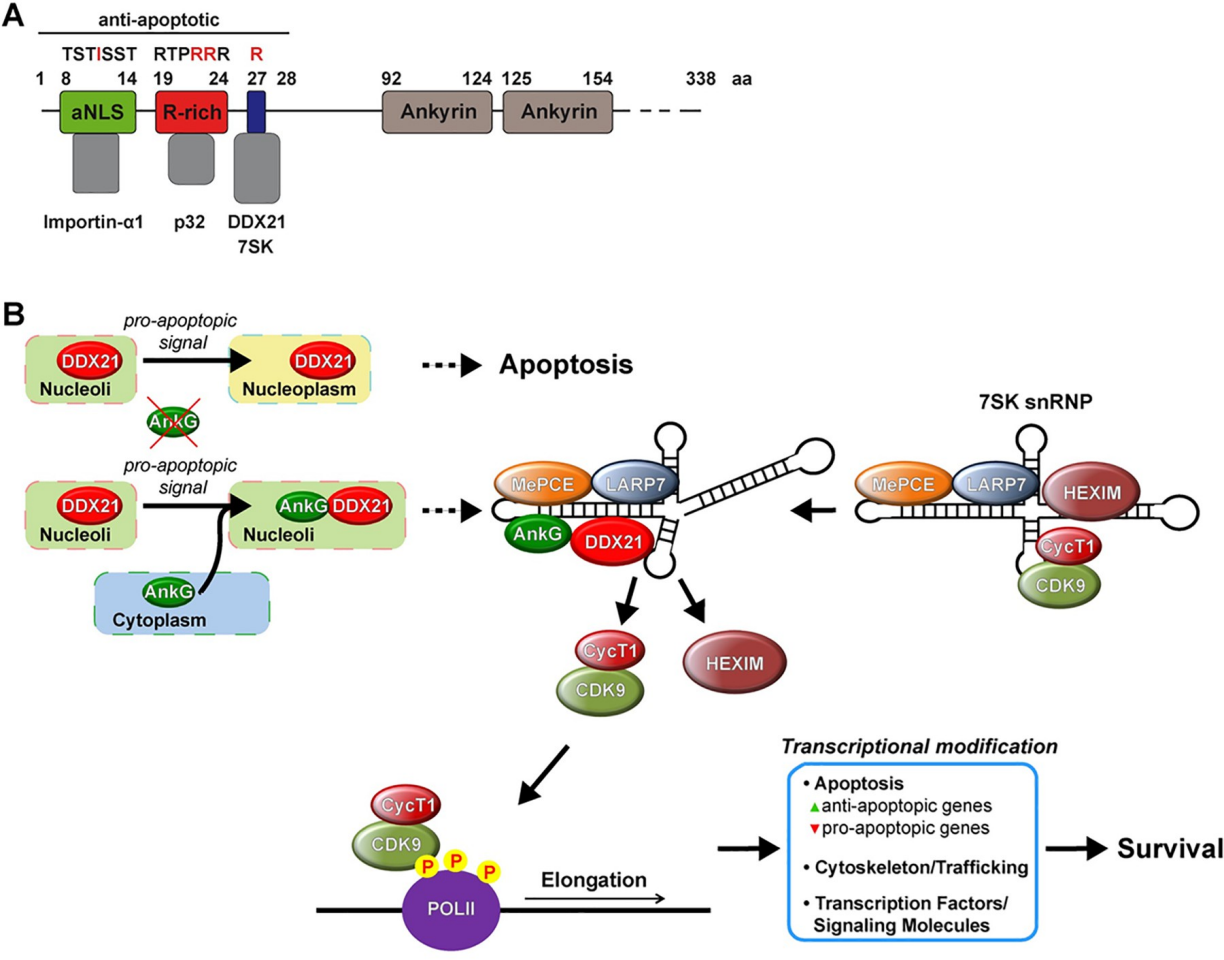
Schematic model of AnkG-mediated modulation of 7SK snRNP activity. (A) Schema of AnkG activity. In addition to the properties mentioned in Fig 1, AnkG interact with the host cell protein DDX21 and with the host cell RNA 7SK. The binding is prevented by mutation of amino acid 27. (B) In the absence of AnkG DDX21 migrates into the nucleoplasm after apoptosis induction. In the presence of AnkG an apoptotic signal results in migration of AnkG into the host cell nucleoli where it binds DDX21. AnkG-DDX21 interacts with the 7SK RNA. This induces the release of P-TEFb (comprising CDK9 and Cyclin T1) and HEXIM from the 7SK snRNP. Released CDK9 phosphorylates NELF, DSIF and the C-terminal domain of Pol II. These promoter-proximal phosphorylation events allow Pol II pause release and transcription of target genes. The presence of AnkG results in transcriptional modulation specifically of genes involved in regulation of apoptosis, trafficking or transcription. As consequence, cells containing AnkG will survive a pro-apoptotic signal.

In summary, nuclear-localized AnkG binds to both, DDX21 and the 7SK RNA, leading to release of CDK9 and reprogramming of host cell transcription. Thus, the *C*. *burnetii* T4SS effector protein AnkG mediates its activity by binding to and influencing the function of the 7SK RNA, a host cell regulatory non-coding RNA (Fig 13).

As DDX21 as well as CDK9 are essential for AnkG activity, we hypothesize that AnkG augments the DDX21-induced conformational changes of the 7SK RNA, which results in release of CDK9 [22]. The CDK9-mediated induction of host cell transcription might convey efficient intracellular replication of *C*. *burnetii* and the ability to prevent host cell apoptosis (Fig 13). This hypothesis is further supported by our data showing that AnkG influences transcription also during infection (Fig 10A and 10B), and that DDX21 as well as AnkG are crucial for efficient anti-apoptotic activity and CCV formation during *C*. *burnetii* infection (Fig 12B and 12C). Interestingly, we observed restored anti-apoptotic activity of the complementation of the Δ*ankG* mutant (Figs 9A and 12B), but the Δ*ankG*::AnkG strain did not restore the replication defect of the Δ*ankG* mutant (Fig 12A). These data suggest that the reduced number of intracellular bacteria did not contribute to the reduced ability to inhibit apoptosis. More experiments will be necessary to verify this hypothesis. Thus, different time points of infection or infection with higher MOIs for the mutant than the wild-type could be used to equal the bacterial loading and to determine the role of bacterial number and/or AnkG for the anti-apoptotic activity of *C*. *burnetii*. Furthermore, the question whether ectopic expression of GFP-NLS-AnkG or different AnkG mutants (mutants that only contain the anti-apoptotic region or mutants lacking the anti-apoptotic region or mutants that are unable to bind to different host cell interaction partners) might restore the defect of the Δ*ankG* mutant in anti-apoptotic activity and CCV formation should be addressed. This might help to dissect the different activities of AnkG during infection.

The 7SK RNA is not the only host cell RNA shown to bind to AnkG (Table 1). Thus, further research is required to fully understand how AnkG influences host cell RNAs to enable bacterial intracellular survival and replication. We have also identified additional host proteins as potential binding partners of AnkG (S1 Fig). The role of these host proteins for AnkG function has to be determined. As AnkG binds several host proteins and host RNAs, the question arises whether the binding of AnkG to a specific protein or RNA is direct or indirect and mediated via protein-complexes or RNPs. Thus, it will be crucial to demonstrate direct binding by GST-pull down or yeast two hybrid experiments. Furthermore, the role of AnkG during infection in macrophages, the primary target cells of *C*. *burnetii*, has to be investigated in more detail.

## Methods

### Reagents, cells and bacterial strains

Chemicals were purchased from Sigma Aldrich unless indicated otherwise. Xtreme Gene 9 transfection reagent were purchased from Roche. Polyethylenimine was used as transfection reagent (Polyscience Inc.). Staurosporine was from Cell Signaling. HEK293T cells (human embryonic kidney cells), HEK293T LARP7^-/-^ cell lines (1–11 and 3–6) and the corresponding control cells; HeLa cells (human epithelial cervix cells), HeLa Bax/Bak ^-/-^, HeLa Bcl-X_L_ overexpression and the corresponding control cells were cultured at 37°C and 5% CO_2_ in Dulbecco’s modified Eagle’s medium (Invitrogen) containing either 5% (for infection) or 10% heat-inactivated fetal bovine serum (Biochrom). THP-1 cells (human monocytic cell line) were cultured at 37°C and 5% CO_2_ in RPMI 1640 (Invitrogen) containing 10% heat-inactivated fetal bovine serum (Biochrom). *E*. *coli* strains DH5α and DH10β were cultivated in Luria-Bertani (LB) broth. If appropriate, selected antibiotic (kanamycin or ampicillin) was added. *C*. *burnetii* (Nine Mile RSA439 phaseII, 1x10^6^/ml) inoculated ACCM-2 or ACCM-D medium (Sunrise Science Products) was incubated at 37°C, 2.5% O_2_ and 5% CO_2_ for 5 days.

### GFP-Trap

A modified protocol from Schäfer *et al*., 2017 was used [16]. In short, HEK293T cells were co-transfected with the respective plasmids. After 24 h cells were lysed in lysis buffer (10 mM Tris/HCl [pH 7.5], 150 mM NaCl, 0.5 mM EDTA, 0.5% Nonidet-P40) for 30 min on ice with pipetting every 10 minutes. Afterwards, lysates were centrifuged for 10 min, 20,000 x g at 4°C and the supernatants were incubated with GFP-Trap_MA beads (ChromoTek) for 2 h at 4°C. The beads were washed three times with washing buffer (10 mM Tris/HCl [pH 7.5], 150–200 mM NaCl, 0.5 mM EDTA) and proteins were resuspended in Laemmli buffer and boiled at 95°C for 5 min. Analysis was performed by immunoblotting.

### Immunoblotting

Proteins were separated by SDS-PAGE on a Bolt Bis-Tris Plus 4–12% gradient gel (Thermo Fisher Scientific) at 180V for 30 min and transferred to a PVDF membrane (Millipore). Proteins were detected using specific primary antibodies against CDK9 (Cell Signaling), DDX21 (Abcam), GFP (Thermo Fisher Scientific), LARP7 (Abcam), bacterial HSP60 (Enzo), actin (Sigma), GAPDH (Cell Signaling), HA (Roche) and HRP-conjugated secondary antibodies (Dianova). Visualization occurred by a chemiluminescence detection system (Thermo Fisher Scientific).

### Indirect immunofluorescence

Infected or transfected HeLa cells seeded in a 24 well plate on coverslips were fixed with 4% paraformaldehyde (Alfa Aesar) in PBS (Biochrom) for 20 min at room temperature, permeabilized with ice-cold methanol for 30 s, quenched with 50 mM NH_4_Cl (Roth) in blocking buffer (PBS/ 5% goat serum) (Life Technologies) for 30 min at room temperature. The coverslips were incubated with anti-DDX21 (Abcam), anti-LAMP2 (DSHB), anti-HA.11 (Covance), and anti-*C*. *burnetii* diluted in blocking buffer, washed with PBS and incubated with secondary Alexa Fluor labelled antibodies Alexa 488 and Alexa 594 (Dianova) in blocking buffer. The cells were mounted using ProLong Diamond with DAPI (Thermo Fisher Scientific). Analysis was performed using the Carl Zeiss LSM 700 Laser Scanning Confocal Microscope or the Zeiss Axio Observer Epifluorescence Microscope.

### RNA immunoprecipitation (RIP)

HEK293T cells were transfected with plasmids for the proteins of interest. After 24 h cells were used for RIP-assay utilizing EZ-Magna Nuclear RIP (Cross-Linked) Nuclear RNA-Binding Protein Immunoprecipitation Kit (Millipore) according to manufacturer’s protocol. Analysis was performed by qRT-PCR.

### qRT-PCR

RNA was used as template for cDNA synthesis with oligo-(dT)-primers (Thermo Fisher Scientific) using SuperScriptII reverse transcriptase (Thermo Fisher Scientific) as recommended by manufacturer. RNA was diluted to achieve a final concentration of cDNA of 5 ng/μl. Quantitative PCR was performed in a 384-well plate using SybrGreen qPCR Mix (Thermo Fisher Scientific) with primers a476/a477 (7SK RNA), a746/a747 (EEF1A2), a1191/a1192 (EGR1), a1195/a1196 (KLF4), a1199/a1200 (JMJD1C), a1203/1204 (CREB3L1), a1211/a1212 (RTN4RL2) and 727/728 (GAPDH). All used primers are shown in Table 4.

**Table 4:** Used primers.

Number	Sequence 5´- 3´	Restriction enzyme
329	5‘-CCAAGATCTCTATGAGTAGACGTGAGACTCC-3‘	BglII
340	5‘-CCGGTACCTCACCGAGGACTAGACAGA-3‘	KpnI
374	5‘-ATGCAATTGTTGTTGTTAACTTG-3‘	-
420	5‘-CCCGGTATGTACCCATACGATGTTCCA-3‘	-
618	5‘-ACAGGATGTCCTGATCGGCTCAATC-3‘	-
619	5‘-CCGAGAAGGAGCTTGAGCCGAAAA-3‘	-
727	5‘-AGGGCTGCTTTTAACTCTGGT-3‘	-
728	5‘-CCCCACTTGATTTTGGAGGGA-3‘	-
746	5‘-CCACTCAGATCTCTCCTAAGAAGAAAAGGAAGGTTAGTAGACGTG AGACTCCCACTAGCACAA-3‘	BglII
1121	5’-CGGGGTACCATGCCGGGAAAACTCCGTA-3’	KpnI
1122	5’-CGGGGTACCTTATTGACCAAATGCTTTACTGA-3’	KpnI
a373	5‘-GGGGTACCCTCCAGCTGCCTCCCCTGGAGAGACTGACCCTGAGTA GACGTGAGACTCCCACTAGC-3‘	KpnI
a476	5’-TCTTCGGTCAAGGGTATACGAGTAG-3’	-
a477	5‘-TACAAATGGACCTTGAGAGCTTGT-3‘	-
a603	5‘-GCGAATTCGG ATGGAAACTGAAAGTGGAAATCAGG-3‘	EcoRI
a604	5‘-GCGGTACCTCAATCATATTCAGAAAATCTTATATGTTTAC-3‘	KpnI
a630	5´-GATCTTGAAGTACCTATTCCGA-3´	-
a631	5´-TGGAACTAGATTTCACTTATCTG-3´	-
a678	5‘-GGGAATTCTCATTTGAGGCTCAATCTCCTTCTCG-3‘	EcoRI
a679	5‘-GGGAATTCTCATGATCGGCTCAATCTCCTTCTCG-3‘	EcoRI
a680	5‘-GGGAATTCTCATTTTCGGCTCAAGCTCCTTCTCG-3‘	EcoRI
a681	5‘-GGGAATTCTCATTTTCGGCTGTTTCTCCTTCTCG-3‘	EcoRI
a682	5‘-GGGAATTCTCATTTTCGGGCCAATCTCCTTCTCG-3‘	EcoRI
a686	5’-GGGAATTCTCATGATCGGCTCAAGCTCCTTCTCG-3’	EcoRI
a700	5´-TAGGGCCCCACAGCTAACACCACGTC-3´	ApaI
a701	5´-TTGGGCCCTCACCGAGGACTAGACAG-3´	ApaI
a702	5´-CTAGATTTAAGAAGGAGATCTGCAGATGAGTAGACGTGAGACTCC-3´	-
a703	5´- GGAGTCTCACGTCTACTCATCTGCAGATCTCCTTCTTAAATCTAG-3´	-
a746	5‘-CGACAACGTCGGCTTCAATGTGAAG-3’	-
a747	5‘-CTGGGAGGTGAACTGAGCAGCC-3’	-
a995	5‘-GGGAATTCTCATTTTTCGCTCAATCTCCTTCTCG-3‘	-
a996	5‘-GGGAATTCTCATTTTTTGCTCAATCTCCTTCTCG-3‘	-
a997	5‘-GGGAATTCTCATTTTTGGCTCAATCTCCTTCTCG-3‘	-
a1191	5‘-CTACGAGCACCTGACCGCAGAG-3’	-
a1192	5‘-CAAGCTGAAGAGGGGCTCGGG-3’	-
a1195	5‘-CACACAGGTGAGAAACCTTACCACTG-3’	-
a1196	5‘-GGCGGTGCCCCGTGTGTTTAC-3’	-
a1199	5‘-TGGAAACAAGGACAGCCTGCAGTG-3’	-
a1200	5‘-GCAGTTCAGGAGATCAGCTTGGTG-3’	-
a1203	5‘-CACCAAGTACCTGAGTGAGGCC-3’	-
a1204	5‘-GGAGAGTTTGATGGTGGTGTTGGG-3’	-
a1211	5‘-GCGGCTGCAGTCGCTGCATTTG-3’	-
a1212	5‘-CAAGTCATCCTGTAGGTGGAGCAG-3’	-

### Nuclear fragmentation assay–transfected cells

HeLa cells were seeded on coverslips in 24-well plates. After 24 h, cells were transfected with the plasmids indicated. Twenty-four hours post-transfection, the cells were incubated with staurosporine (0.1μM) for 4 h at 37°C and 5% CO_2_. The cells were fixed with 4% paraformaldehyde (Alfa Aesar) in PBS for 20 min at room temperature, permeabilized with ice-cold methanol for 30 s, quenched with 50 mM NH_4_Cl (Roth) in PBS for 15 min at room temperature. Finally, the cells were mounted using ProLong Diamond with DAPI (Thermo Fisher Scientific). The number of fragmented nuclei were determined using an epifluorescence microscope. To evaluate the impact of CDK9 inhibition, 200 nM THAL SNS 032 (TOCRIS) was added 24 h post transfection for 4 h.

### Nuclear fragmentation assay–infected cells

HeLa cells (5x10^4^) were seeded 24 h before infection on coverslips in a 24-well plate. Cells were infected with *C*. *burnetii* at a MOI of 200 by centrifugation (900 rpm, 10 min without brake). THP-1 cells (1x10^5^ in 500 μl) were seeded 48 h before infection on coverslips in a 24-well plate. Phorbol 12-myristate-13-acetate (PMA) was added 24 h before infection in 500 μl new medium at a final concentration of 200 nM to allow differentiation of the cells toward macrophages. The PMA containing medium was removed and replaced by fresh RPMI 1640/ 10% FCS 4 h prior infection with *C*. *burnetii* at a MOI of 50 by centrifugation (900 rpm, 10 min without brake). Twenty-four hours post-infection all cells were washed three times with PBS and supplemented with fresh medium. Seventy-two hours post-infection the cells were incubated with staurosporine (0.1 μM for HeLa cells and 0.5 μM for THP-1 cells) for 4 h at 37°C and 5% CO_2_ and prepared for indirect immunofluorescence with anti-LAMP2 and anti-*C*. *burnetii* as described above. The percentage of fragmented or condensed nuclei of infected cells were determined using an epifluorescence microscope.

### DDX21 siRNA knock-down

DDX21 siRNA and non-target siRNA as well as transfection reagent were purchased from Dharmacon. The respective amount was first diluted in 1x siRNA buffer. Subsequently, the siRNA solution was mixed with an equal amount of DMEM medium without serum containing DharmaFECT 1, and incubated at room temperature for 20 min. The siRNA-DharmaFECT-solution was mixed with an equal amount of cell solution (5x10^4^ for 24-well, 1x10^5^ for 6-well) and seeded on respective plate. Transfection with DNA was performed 24 h post siRNA transfection. Infection with different *C*. *burnetii* strains at MOI of 200 was done 24 h before siRNA transfection and subsequent analysis was performed 48 h post siRNA treatment (equals 72 h post infection).

### RNA sequencing and pathway analysis

For RNA-sequencing HEK293T cells were transfected with GFP or GFP-NLS-AnkG, sorted in cooperation with the FACS-CORE unit Erlangen, and RNA was isolated with TriFast reagent (VWR) according to manufacturer’s instructions. Isolated RNA was treated with DNase I (Qiagen) for 30 min at 37°C and again purified using TriFast reagent. The sequencing of the samples was performed by GATC (Eurofins). The reads were then aligned to the human genome (hg38 / GRC38, UCSC) using Bowtie2 version 2.3.3.1 and subsequently samtools version 0.1.18 to generate bam files. All raw data is available at GEO (GSE185428). The resulting bam files were then analyzed using Cufflinks [71] and Cuffdiff [72] against the GENCODE primary assembly annotation version 37. The correlation between the samples was controlled using deepTools 3.3.0 MultiBamSummary [73]. The Cuffdiff results were filtered for a minimal FPKM value of 1 in at least one sample. As cutoffs for significantly differentially expressed genes, a minimal fold change of 1.5 and p-value of 0.001 were chosen. The volcano plots were generated from the Cuffdiff results using ggplot2 (https://ggplot2.tidyverse.org) in R-Studio. The gene ontology (GO) analysis was performed using the online tool gprofiler (https://biit.cs.ut.ee/gprofiler/gost). To annotate different pathways, genes included in specific GO terms were obtained from the QuickGO website (https://www.ebi.ac.uk/QuickGO), where the genes for human were downloaded including child terms. The expression heatmap of the differentially expressed genes was created over the results of Cufflinks analysis for all samples using seaborn clustermap (https://doi.org/10.21105/joss.03021) in Python 3. For RIP-Seq analysis, the RIP assay (see above) was performed prior to RNA isolation and DNase digestion. Gene Ontology (GO) analysis was performed using Gorilla online tool.

### Generation of *C*. *burnetii* Δ*ankG* and the complemented strain

*C*. *burnetii* Nine Mile phase II were electroporated with 10μg pJC-CAT::*ankG*-5´3´-lysCA as previously described [17]. To generate an AnkG complemented strain the deficient mutant strain was co-electroporated with 20μg pMiniTn7T-ArgGH-ProC-AnkG and 10μg pTnS2::1169^P^-*tnsABCD* as described before [74]. Tn7 transposase mediated integrants were selected by culturing the bacteria in ACCM-D media lacking lysine and lacking arginine, but containing citrulline for 5 days as previously described [75]. The diluted culture was spread on 0.25% ACCM-D agarose without lysine and arginine, but containing citrulline for 10 days. Individual clones were picked and expanded in ACCM-D medium lacking lysine and arginine but containing citrulline.

### Analysis of *C*. *burnetii* infected HeLa cells

HeLa cells (5x10^4^) were seeded on coverslips in a 24-well plate. Twenty-four hours post-seeding cells were infected with *C*. *burnetii* with an MOI of 200 for 12, 36, and 60 h. The cells were fixed and immunofluorescence (see above) was performed.

### CDK9 release assay

To determine CDK9 release, 1.5x10^6^ HEK293T cells were seeded in 10 cm dishes for 24 h. Cells were co-transfected with plasmids encoding GFP-tagged LARP7 and HA-tagged AnkG variants. Twenty-four hours post-transfection a GFP-trap (see above) was performed. The GFP-trap precipitates were analyzed by immunoblot analysis (see above) using anti-GFP, anti-HA, and anti-CDK9 antibodies. Immunoblots were used for densitometry by ImageJ. Peak areas were calculated and CDK9 signal was normalized to the GFP signal using artificial units.

### Software

For significance calculations, an unpaired Student’s T-test in MS Excel was utilized. Volcano plot and heat maps were generated using R. Densitometry was performed with ImageJ. Sequence analysis was done with LaserGene package.

### Statistical analysis

An unpaired Student´s t-test was used for statistical analysis.

### Plasmid construction

Plasmids used in this study are listed in Table 5. For plasmid construction, DNA fragments were amplified from HeLa cell cDNA (eukaryotic genes) or from the plasmids pEGFP-AnkG, pCMV-HA-AnkG and pSB3C5-ProC-GFP utilizing primers listed in Table 4. Amplicons as well as the respective vector plasmid were digested using indicated restriction enzymes (Table 4) for 2 h to overnight. Subsequently, ligation was performed using T4 ligase (Thermo Fisher Scientific) and plasmids were transformed in *E*. *coli* DH5α or DH10β. Positive clones were identified by PCR using vector based primers (618/619 for pEGFP, 374/420 for pCVM-HA, and a630/a631 for pMiniTn7T-ArgGH). For construction of the AnkG complementation plasmid pMiniTn7T-ArgGH-ProC-AnkG an overlap extension PCR approach using Q5 Hot start PCR polymerase (NEB) was utilized as described elsewhere [76]. The template pSB3C5-ProC-GFP and PCR primers a700/ a703 (Table 4) were used to generate fragment A containing a 5´-ApaI restriction site, the insulated bacterial promoter ProC [33] and an overlap to the AnkG gene. The template pCMV-HA-AnkG [15] and PCR primers a702/ a701 (Table 4) were used to generate fragment B containing an overlap to the ProC promoter, AnkG and a 3´-ApaI restriction site. Fragments A and B were used as templates in the final PCR with the pimers a700/ a701 resulting in the fused product ProC-AnkG. Sequencing was performed using Macrogen sequencing service.

**Table 5:** Used plasmids.

Plasmid	Primers	Reference
pCMV-HA	-	Clontech
pCMV-HA-AnkG	-	Lührmann *et al*. (2010) [15]
pCMV-HA-AnkG_70-338_	-	Lührmann *et al*. (2010) [15]
pCMV-HA-DDX21	1121 / 1122	this study
pCMV-HA-LARP7	a603 / a604	this study
pCMV-HA-NES-AnkG	a373 / 340	this study
pCMV-HA-NLS-AnkG	746 / 340	this study
pEGFP	-	Clontech
pEGFP-AnkG	-	Lührmann *et al*. (2010) [15]
pEGFP-AnkG_1-28_	-	Schäfer *et al*. (2020) [17]
pEGFP-AnkG_1-28 R24S_	329 / a680	this study
pEGFP-AnkG_1-28 L25N_	329 / a681	this study
pEGFP-AnkG_1-28 S26A_	329 / a682	this study
pEGFP-AnkG_1-28 R27S_	329 / a678	this study
pEGFP-AnkG_1-28 R27E_	329 / a995	this study
pEGFP-AnkG_1-28 R27K_	329 / a996	this study
pEGFP-AnkG_1-28 R27Q_	329 / a997	this study
pEGFP-AnkG_1-28 K28S_	329 / a679	this study
pEGFP-AnkG_1-69_	-	Eckart *et al*. (2014) [14]
pEGFP-AnkG_70-338_	-	Eckart *et al*. (2014) [14]
pEGFP-DDX21	1121 / 1122	this study
pEGFP-LARP7	a603 / a604	this study
pEGFP-NLS-AnkG	746 / 340	this study
pJC-CAT::ankG-5´3´-lysCA		(Schäfer *et al*., 2020) [17]
pSB3C5-ProC-GFP		(Davis *et al*., 2011) [33]
pTnS2::1169^P^-*tns ABCD*		(Beare *et al*., 2011) [77]
pMiniTn7T-ArgGH-ProC-AnkG	a700/a701	this study

## Supporting information

S1 Fig AnkG might interact with several host cell nuclear proteins. HEK293T cells were transiently co-transfected with plasmids encoding HA-NLS-AnkG and GFP-tagged host nuclear proteins or GFP. Proteins were precipitated using GFP-trap. Western blot analysis was used to detect AnkG (anti-HA) and GFP or GFP-tagged host nuclear proteins (anti-GFP) in the lysates (Pre-IP) and in the precipitates (IP).
(TIF)

S2 Fig AnkG-DDX21 interaction, mediated by hydrogen bond, is essential for AnkG-driven apoptosis-inhibition. (A) GFP and GFP-tagged AnkG_1-28_-mutations were transiently co-expressed with HA-DDX21 in HEK293T cells. Proteins were precipitated using GFP-trap. Western blot analysis was used to detect AnkG_1-28_ mutations (anti-GFP) and HA-DDX21 (anti-HA) in the lysates (Pre-IP) and in the precipitates (IP). One out of three independent experiments with similar results is shown. (B) GFP, GFP-AnkG_1-28_ or GFP-AnkG_1-28_ mutants were transiently expressed in HeLa cells. The cells were treated with 0.1 μM staurosporine for 4 h, fixed and stained with an antibody against endogenous DDX21. 100 transfected cells each were analyzed for the DDX21 localization. The result of four independent experiments is shown. Error bars indicate ± SD. *** *p*< 0.001.
(TIF)

S3 Fig AnkG induced transcriptional reprogramming. HEK293T cells were transfected with plasmids encoding GFP or GFP-NLS-AnkG. GFP-positive cells were sorted and total RNA was isolated and reverse transcribed in cDNA using SuperScript II reverse transcriptase according to the manufacturer’s protocol. A qRT-PCR was performed with primers amplifying fragments of the indicated genes. The ΔΔCt values were calculated for the fold difference of expression in GFP-NLS-AnkG- versus GFP-expressing cells.
(TIF)

S4 Fig AnkG-mediated alteration of CCV maturation might not depend on its anti-apoptotic activity. (A—D) Bax/Bak double knock-out HeLa cells, Bcl-x_L_ overexpressing HeLa cells and respective control cell lines were infected with *C*. *burnetii* wild-type or an *ankG* deletion strain (Δ*ankG*). At 12 and 60 hours post-infection the cells were fixed stained with antibodies against *C*. *burnetii* and LAMP-2. (A) The infection rate of 100 cells each was determined by epifluorescence microscopy. Shown is the mean of three independent experiments. Error bars indicate ± SD. (B and C) Representative images of Bax/Bak double knock-out HeLa cells and the respective control cell line were used to determine (B) the vacuole number per cell and (C) the size of the CCVs (small, big or giant) from 100 infected cells each at 60 hours post-infection. The experiment was performed three times. Error bars indicate ± SD. ** p< 0.01, *** p<0.001. (D and E) Representative images of Bcl-xL overexpressing HeLa cells and the respective control cell line were used to determine (D) the vacuole number per cell and (E) the size of the CCVs (small, big or giant) from 100 infected cells each at 60 hours post-infection. The experiment was performed three times. Error bars indicate ± SD. ** p< 0.01, *** p<0.001.
(TIF)

S1 Table RNAs identified in the RIP-Seq analysis. The fragments per kilobase million (FPKM) from three independent experiments are shown for each of the identified potential AnkG interacting RNAs.
(XLSX)

S2 Table The gene-ID, the gene label and p-value of all genes differential expressed in cells expressing GFP-NLS-AnkG in comparison to the expression level in cells expressing GFP. (XLSX)
