# Supplementary figures and images for: The Coxiella burnetii T4SS effector protein AnkG hijacks the 7SK small nuclear ribonucleoprotein complex for reprogramming host cell transcription
Source: PLoS Pathog. 2022 Feb 8;18(2):e1010266. doi: 10.1371/journal.ppat.1010266 (PMC8824381; doi:10.1371/journal.ppat.1010266)

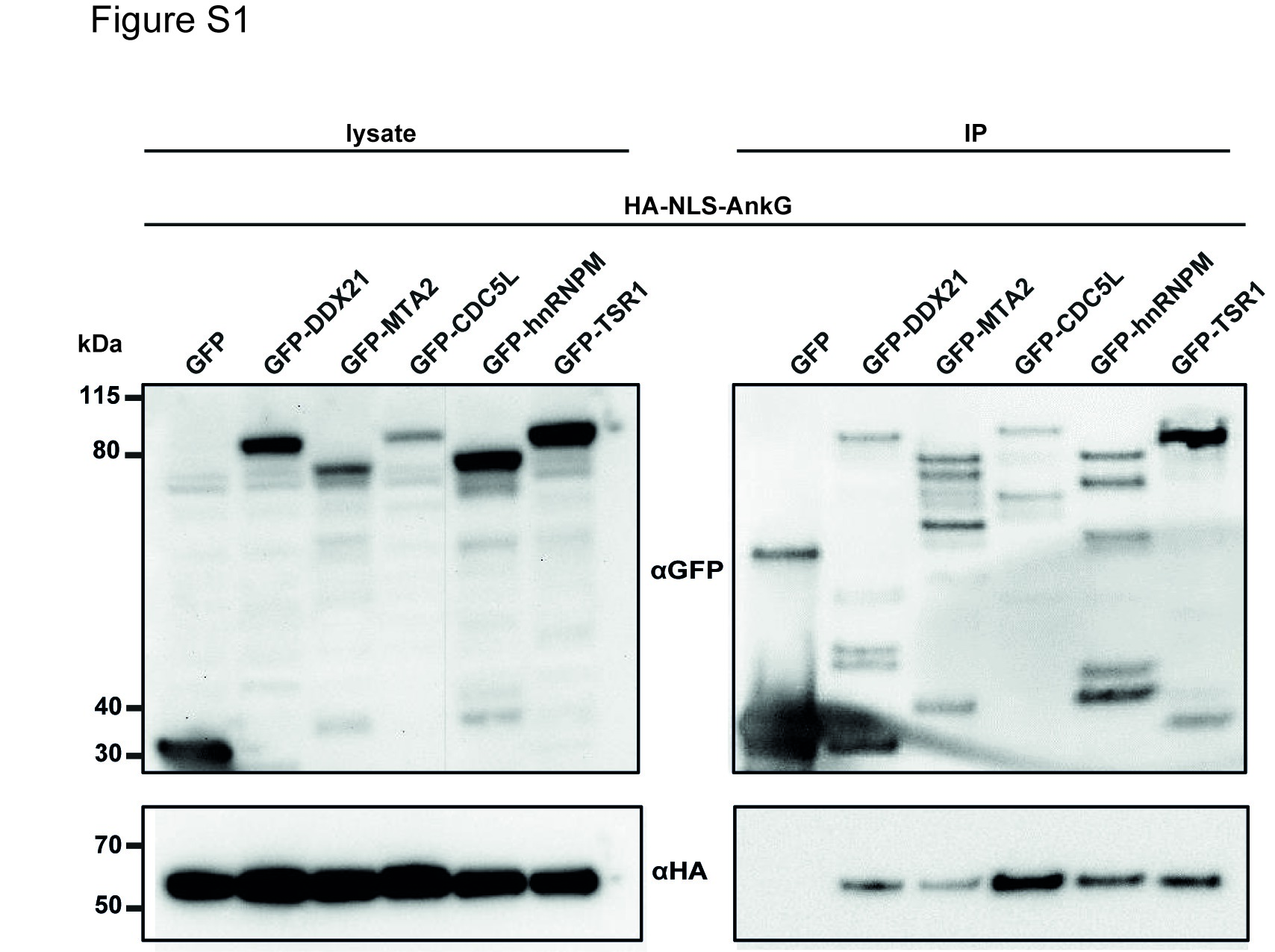

Supplement: S1 Fig — HEK293T cells were transiently co-transfected with plasmids encoding HA-NLS-AnkG and GFP-tagged host nuclear proteins or GFP. Proteins were precipitated using GFP-trap. Western blot analysis was used to detect AnkG (anti-HA) and GFP or GFP-tagged host nuclear proteins (anti-GFP) in the lysates (Pre-IP) and in the precipitates (IP). (TIF) [file ppat.1010266.s001.tif]

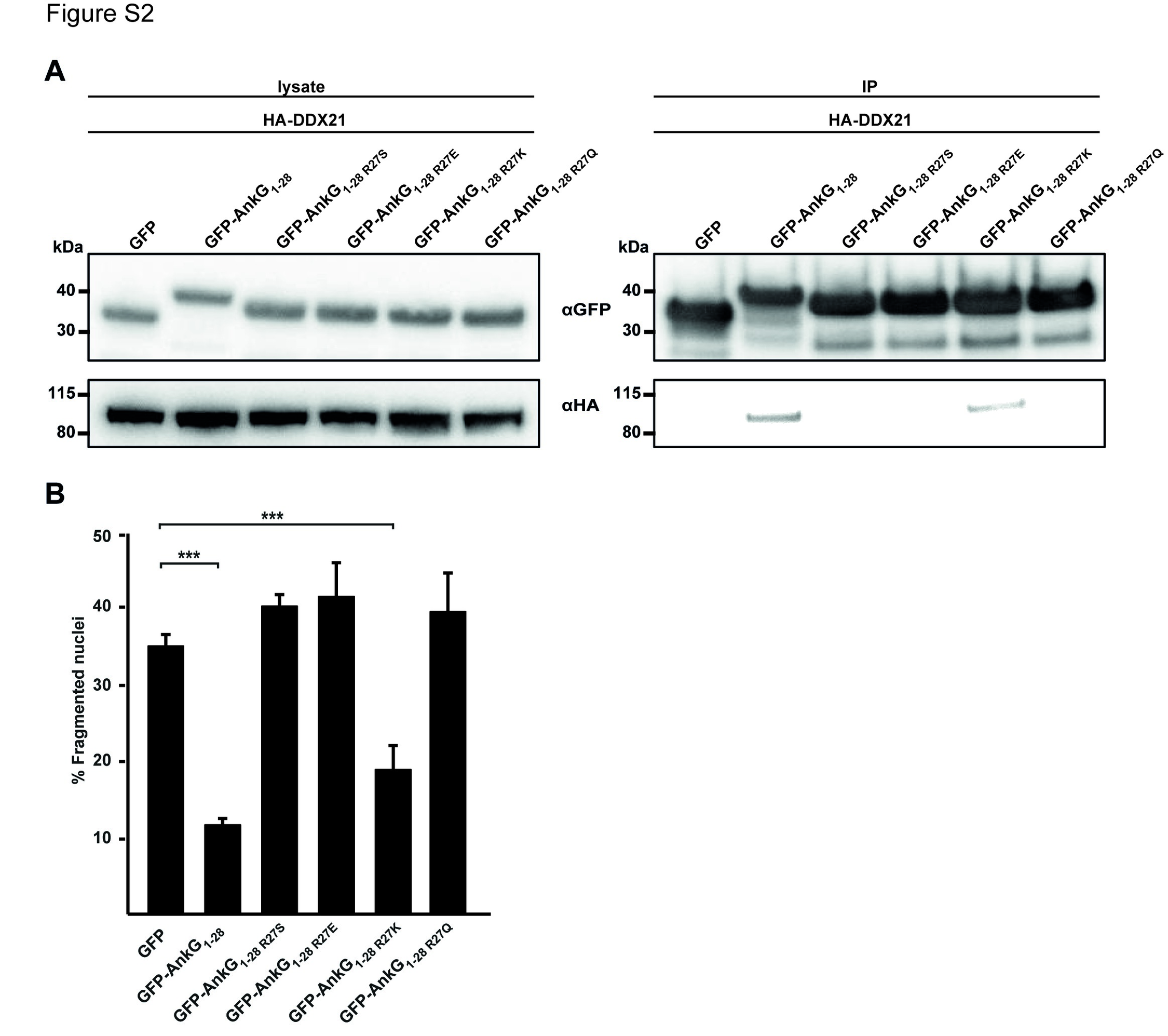

Supplement: S2 Fig — (A) GFP and GFP-tagged AnkG1-28-mutations were transiently co-expressed with HA-DDX21 in HEK293T cells. Proteins were precipitated using GFP-trap. Western blot analysis was used to detect AnkG1-28 mutations (anti-GFP) and HA-DDX21 (anti-HA) in the lysates (Pre-IP) and in the precipitates (IP). One out of three independent experiments with similar results is shown. (B) GFP, GFP-AnkG1-28 or GFP-AnkG1-28 mutants were transiently expressed in HeLa cells. The cells were treated with 0.1 μM staurosporine for 4 h, fixed and stained with an antibody against endogenous DDX21. 100 transfected cells each were analyzed for the DDX21 localization. The result of four independent experiments is shown. Error bars indicate ± SD. *** p< 0.001. (TIF) [file ppat.1010266.s002.tif]

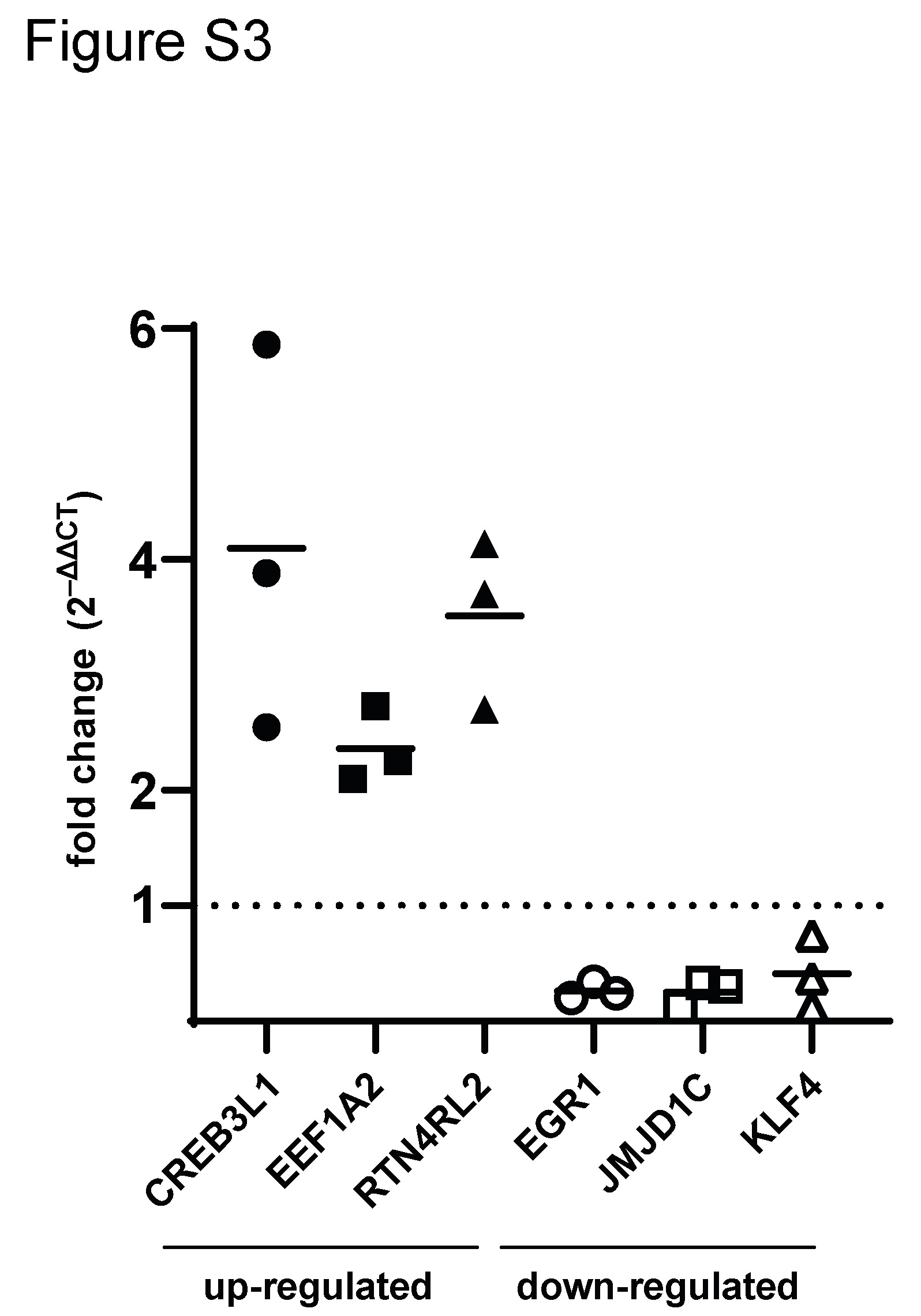

Supplement: S3 Fig — HEK293T cells were transfected with plasmids encoding GFP or GFP-NLS-AnkG. GFP-positive cells were sorted and total RNA was isolated and reverse transcribed in cDNA using SuperScript II reverse transcriptase according to the manufacturer’s protocol. A qRT-PCR was performed with primers amplifying fragments of the indicated genes. The ΔΔCt values were calculated for the fold difference of expression in GFP-NLS-AnkG- versus GFP-expressing cells. (TIF) [file ppat.1010266.s003.tif]

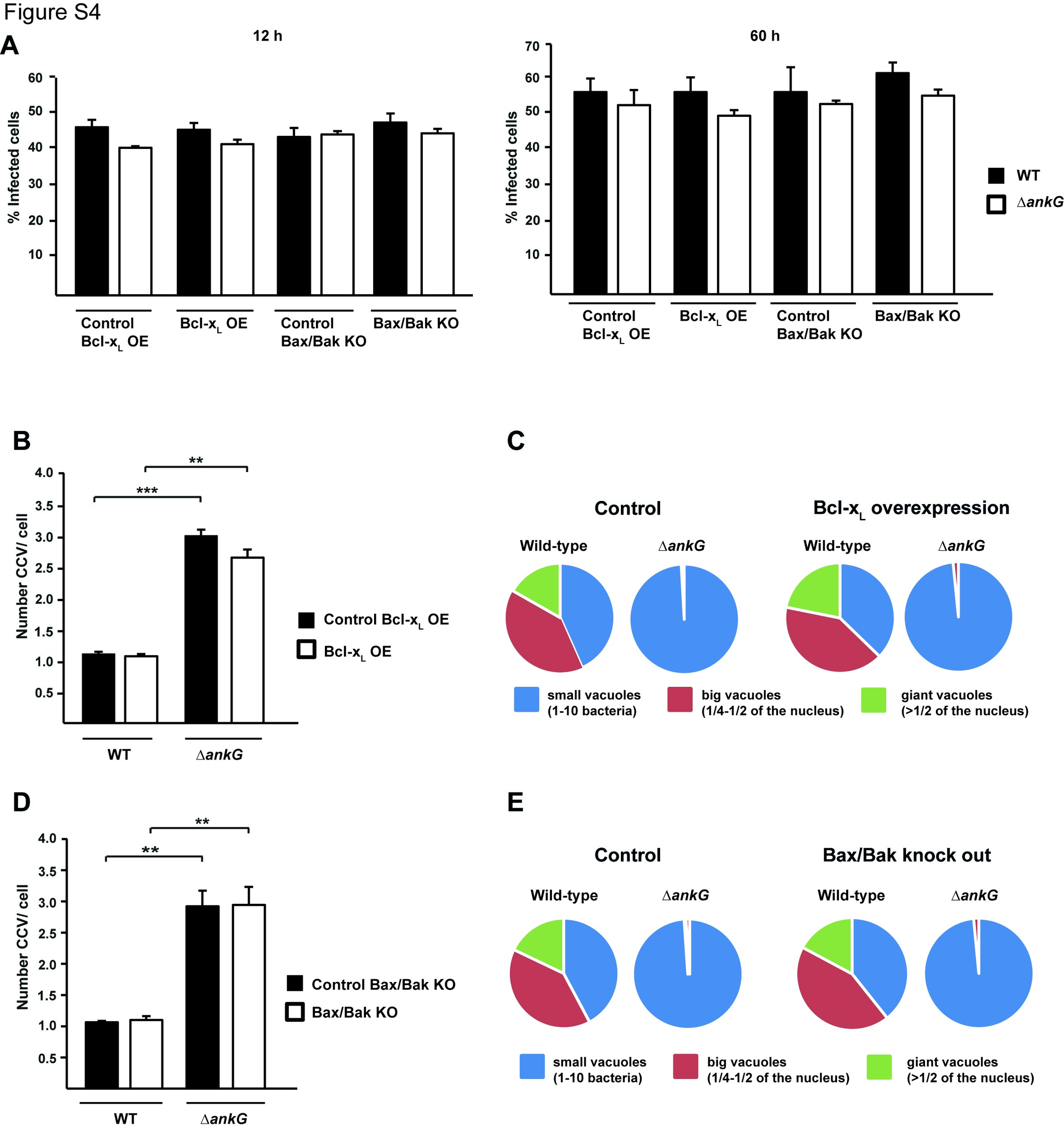

Supplement: S4 Fig — (A—D) Bax/Bak double knock-out HeLa cells, Bcl-xL overexpressing HeLa cells and respective control cell lines were infected with C. burnetii wild-type or an ankG deletion strain (ΔankG). At 12 and 60 hours post-infection the cells were fixed stained with antibodies against C. burnetii and LAMP-2. (A) The infection rate of 100 cells each was determined by epifluorescence microscopy. Shown is the mean of three independent experiments. Error bars indicate ± SD. (B and C) Representative images of Bax/Bak double knock-out HeLa cells and the respective control cell line were used to determine (B) the vacuole number per cell and (C) the size of the CCVs (small, big or giant) from 100 infected cells each at 60 hours post-infection. The experiment was performed three times. Error bars indicate ± SD. ** p< 0.01, *** p<0.001. (D and E) Representative images of Bcl-xL overexpressing HeLa cells and the respective control cell line were used to determine (D) the vacuole number per cell and (E) the size of the CCVs (small, big or giant) from 100 infected cells each at 60 hours post-infection. The experiment was performed three times. Error bars indicate ± SD. ** p< 0.01, *** p<0.001. (TIF) [file ppat.1010266.s004.tif]
